# The Drosophila Y Chromosome Affects Heterochromatin Integrity Genome-Wide

**DOI:** 10.1093/molbev/msaa082

**Published:** 2020-03-25

**Authors:** Emily J Brown, Alison H Nguyen, Doris Bachtrog

**Affiliations:** Department of Integrative Biology, University of California Berkeley, Berkeley, CA

**Keywords:** Drosophila, heterochromatin, chromatin sink, sex chromosomes, Y chromosome

## Abstract

The Drosophila Y chromosome is gene poor and mainly consists of silenced, repetitive DNA. Nonetheless, the Y influences expression of hundreds of genes genome-wide, possibly by sequestering key components of the heterochromatin machinery away from other positions in the genome. To test the influence of the Y chromosome on the genome-wide chromatin landscape, we assayed the genomic distribution of histone modifications associated with gene activation (H3K4me3) or heterochromatin (H3K9me2 and H3K9me3) in fruit flies with varying sex chromosome complements (X0, XY, and XYY males; XX and XXY females). Consistent with the general deficiency of active chromatin modifications on the Y, we find that Y gene dose has little influence on the genomic distribution of H3K4me3. In contrast, both the presence and the number of Y chromosomes strongly influence genome-wide enrichment patterns of repressive chromatin modifications. Highly repetitive regions such as the pericentromeres, the dot, and the Y chromosome (if present) are enriched for heterochromatic modifications in wildtype males and females, and even more strongly in X0 flies. In contrast, the additional Y chromosome in XYY males and XXY females diminishes the heterochromatic signal in these normally silenced, repeat-rich regions, which is accompanied by an increase in expression of Y-linked repeats. We find hundreds of genes that are expressed differentially between individuals with aberrant sex chromosome karyotypes, many of which also show sex-biased expression in wildtype Drosophila. Thus, Y chromosomes influence heterochromatin integrity genome-wide, and differences in the chromatin landscape of males and females may also contribute to sex-biased gene expression and sexual dimorphisms.

## Introduction

**Fig. 1.**
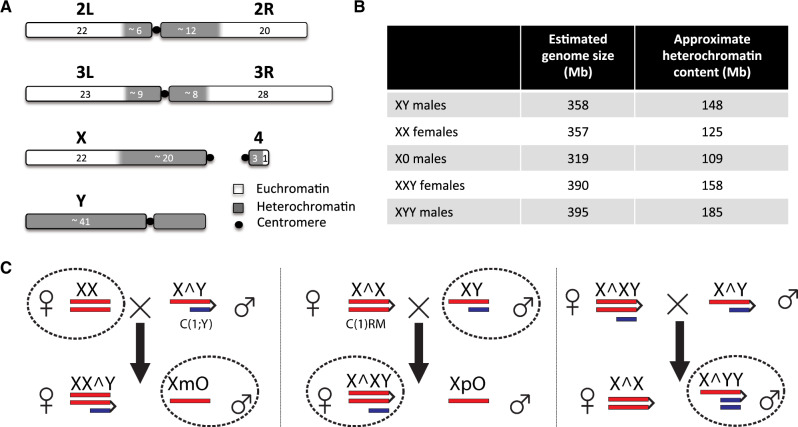
Chromosome structure of *Drosophila melanogaster* and crossing scheme utilized. (*A*) The left and right arms of chromosomes 2 (2L, 2R) and 3 (3L, 3R), the small chromosome 4 (the dot chromosome), and the sex chromosomes X and Y are shown (adapted from Hoskins et al. [2002]). The numbers correspond to approximate lengths in megabases but will differ among Drosophila strains. (*B*) Flow cytometry estimates of the mean diploid genome size of the five karyotypes investigated (based on three replicate measures). The approximate heterochromatin content for the strains investigated is indicated, assuming that the euchromatic size is constant for all chromosomes (i.e., 232 Mb for flies with 2 X chromosomes, and 210 Mb for flies with a single X chromosome, see *A*). (*C*) Crossing scheme utilized to obtain X0 and XYY males, and XXY females (only sex chromosomes are shown). Wildtype Canton-S males and females were crossed to the 2549 strain whose females have C(1)RM and males have C(1;Y). Circled karyotypes were used for the analyses.

The Drosophila Y is a degenerated, heterochromatic chromosome with only a few functional genes, primarily specialized in male reproductive function (Gatti and Pimpinelli 1983; Carvalho et al. 2000, 2001; Carvalho 2002). However, the *Drosophila melanogaster* Y is about 40 Mb in size and accounts for ∼20% of the male haploid genome (Gatti and Pimpinelli 1983; Hoskins et al. 2002) (fig. 1*A*). Most of the Y chromosome is composed of repetitive satellite DNA, transposable elements (TEs), and ribosomal DNA (rDNA) blocks (Bonaccorsi and Lohe 1991), and it is transcriptionally silenced through heterochromatin formation (Elgin and Reuter 2013). Despite harboring only a few genes, natural variation on the Y chromosome is associated with variation in several traits, including male fitness (Chippindale and Rice 2001) and position effect variegation (PEV), that is, the ability of spreading heterochromatin to induce partial silencing of reporter genes in some cells, resulting in mosaic expression patterns (Gowen and Gay 1934). More recently, it was found that natural variation on the Y chromosome has substantial effects on regulation of hundreds of protein-coding genes genome-wide (Dimitri and Pisano 1989; Lemos et al. 2008, 2010; Sackton et al. 2011).

The molecular basis of this phenotypic variation is unclear. Single-nucleotide polymorphism in protein-coding genes is low on the Y chromosome (Zurovcova and Eanes 1999; Larracuente and Clark 2013), and it has been proposed that structural variation involving repetitive DNA is responsible for the observed phenotypic effects of different Y chromosomes (Francisco and Lemos 2014). Specifically, most of the highly repetitive Y chromosome is enriched for heterochromatic proteins and repressive histone modifications, and the Y may act as a “heterochromatin sink.” That is, the Y chromosome may sequester core components of the heterochromatin machinery (such as structural proteins or modifying enzymes that play key roles in establishing and maintaining heterochromatin), thereby limiting the ability to silence other repetitive regions of the genome (Henikoff 1996; Francisco and Lemos 2014). Under the heterochromatin sink model, Y chromosomes vary in their ability to sequester heterochromatin components due to variation in the total amount or sequence content of their repetitive sequences (i.e., their repeat content). Protein-coding genes from the *D. melanogaster* Y chromosome are only expressed in germ cells of males, but the effects on global gene expression by different Y chromosomes also occur in XXY females and somatic cells of XY males (Lemos et al. 2008, 2010; Sackton et al. 2011). This observation is consistent with the heterochromatin sink model, where the Y chromosome exerts its effect indirectly by depleting or redistributing chromatin regulators across the genome (Gatti and Pimpinelli 1992). Indeed, PEV assays with different reporter systems have demonstrated that Y-chromosomal DNA suppresses variegation (Gowen and Gay 1934; Dimitri and Pisano 1989). Interestingly, by using a series of cytologically characterized Y chromosome deficiencies and Y fragments, it was shown that Y chromosomes that are cytologically different yet retain similar amounts of heterochromatin are equally effective suppressors, and suppression effect is positively related to the size of the Y-derived DNA (Dimitri and Pisano 1989). This is consistent with the notion that the Y acts as a heterochromatin sink. However, studies to assess the effect of the Y chromosome on heterochromatin formation have been limited to reporter loci through PEV assays (Gowen and Gay 1934), and the global chromatin landscapes of individuals with different amounts of heterochromatic sequence have not yet been directly examined. In particular, studies of PEV do not directly demonstrate changes in the spreading of heterochromatin along the chromosome but infer it from phenotypic effects on reporter genes (Spofford 1976). In addition, most of the variegating rearrangements have not been characterized at the molecular level, and the precise location of their heterochromatic breakpoints has not been determined (Dimitri and Pisano 1989; Gatti and Pimpinelli 1992); it is thus not known whether the different heterochromatic regions are equally effective in inducing variegation. Most importantly, PEV studies do not directly probe the integrity or amount of heterochromatin at repetitive regions that exert PEV through spreading of heterochromatin. Under the heterochromatin sink model, changes in the amount of repetitive DNA should modify the amount of heterochromatin formed at repeats on a global scale. In particular, increasing the amount of repetitive DNA is expected to result in reduced levels of heterochromatin at repetitive regions, since additional repeats should dilute heterochromatic factors that are present in only limited amounts, whereas decreasing the amount of repetitive DNA should have the opposite effect. Here, we test the hypothesis that the Y chromosome acts to modulate heterochromatin integrity and gene expression genome-wide by contrasting the chromatin landscapes and expression profiles of X0 and XYY males and XXY females to that of wildtype *D. melanogaster*.

## Results

### Fly Strains

To compare the chromatin landscape between Drosophila that differ in their sex chromosome karyotype and their amount of repetitive DNA, we set up replicate crosses between *D. melanogaster* stock number 2549 from the Bloomington Stock Center, which has a compound reversed metacentric X chromosome (C(1)RM) or a heterocompound chromosome with the X chromosome inserted between the two arms of the Y chromosome (C(1;Y)), and the wildtype Canton-S stock (fig. 1*C* and supplementary fig. S1, Supplementary Material online). We selected X0 males that contained a maternally transmitted X chromosome (as do wildtype males), and XXY females that contain a wildtype Y chromosome (rather than the C(1;Y) chromosome; see fig. 1*C*). Note that the resulting flies are not isogenic (and it is impossible to create completely isogenic flies using this crossing scheme), but some of the comparisons contrast flies with identical autosomal backgrounds. In particular, our wildtype male and female comparison share the same autosomal genotype (Canton-S), and our X0 males and XXY females both have one autosomal complement from Canton-S and one from the 2549 stock. XYY males inherit 75% of autosomal genes from strain 2549. We also generated X0, XXY, and XYY flies using a different attached X and attached X–Y stock, 4248, crossed to the wildtype Canton-S stock; these flies allowed us to verify our findings on chromatin redistribution in an independent genetic background (see below). To get a rough estimate on the amount of repetitive DNA present in the five karyotypes with different sex chromosome configurations, we used flow cytometry to estimate the genome sizes. Under the assumption that the size of the euchromatic chromosome arms is constant across karyotypes, and using estimates of diploid euchromatic genome sizes of 232 Mb for individuals with two X chromosomes and 210 Mb for individuals with one X chromosome (see fig. 1*A*), we estimated the amount of heterochromatic sequences in each karyotype. As expected, we found a gradient of heterochromatic sequence content per diploid cell for the five karyotypes, with X0 males (∼109 Mb) < XX females (∼125 Mb) < XY males (∼148 Mb) < XXY females (∼158 Mb) < XYY males (∼185 Mb) (supplementary table S1, Supplementary Material online and fig. 1*B*). Independent characterization of repetitive elements using de novo assembly of repeats with dnaPipeTE (Goubert et al. 2015) confirmed the relative abundance of repeats in each karyotype (supplementary table S2 and fig. S2, Supplementary Material online).

### Quantification of Histone Modifications

We aged independent replicates of all flies for 8 days and carried out chromatin immunoprecipitation followed by DNA sequencing (ChIP-seq) on head and thorax tissue using commercial antibodies against three posttranslational histone modifications (H3K4me3, H3K9me2, and H3K9me3; see supplementary table S3, Supplementary Material online, for an overview of all ChIP data sets generated and supplementary table S4 and figs. S3–S6, Supplementary Material online, for general mapping statistics). GC content bias, that is, the dependency of read coverage and GC content found in Illumina sequencing data, can be especially problematic for repetitive DNA analysis since repeated sequences often have extreme GC contents. We corrected for GC content biases in our ChIP-seq experiments for the heterochromatic marks using a method developed by Benjamini and Speed (2012) and implemented by Flynn et al. (2017). We employed a previously described normalization strategy (Li et al. 2014) to compare the genomic distribution and relative levels of chromatin marks across flies with different karyotypes. Specifically, we “spiked in” a fixed amount of chromatin from female third instar *Drosophila miranda* to each *D. melanogaster* chromatin sample prior to ChIP and sequencing. *Drosophila miranda* chromatin served as an internal standard for the immunoprecipitation experiment (supplementary table S5, Supplementary Material online), and the relative recovery of *D. melanogaster* ChIP signal versus *D. miranda* ChIP signal, normalized by their respective input counts, was used to quantify the relative abundance of the chromatin mark in *D. melanogaster* (see Materials and Methods for details; Li et al. 2014). Note that this normalization strategy uses input coverage to account for differences in ploidy levels of sex chromosomes among the different karyotypes investigated and is agnostic to the total genome size of the sample (supplementary fig. S7, Supplementary Material online). *Drosophila miranda* is sufficiently diverged from *D. melanogaster* for sequencing reads to be unambiguously assigned to the correct species: even in repetitive regions, <4% of the reads cross-mapped between species; these regions were excluded from the analysis.

We also used a different normalization strategy to quantify the absolute abundance of the heterochromatic chromatin marks for each *D. melanogaster* karyotype. In particular, the relative recovery of *D. melanogaster* ChIP signal versus *D. miranda* ChIP signal, normalized by their respective input counts, was estimated using a linear regression model (Bonhoureet al. 2014, see Materials and Methods). Overall enrichment patterns and differences among karyotypes are quantitatively similar between the two methods, showing that our inferences are robust to our normalization strategy (supplementary fig. S8*A*, Supplementary Material online). Repetitive regions pose a challenge for mapping with short reads, since one cannot be sure that a particular locus is generating the reads in question if they map to multiple positions. Our study is concerned with the overall behavior of repetitive regions in the genome, and not focused on any particular locus; thus, analyzing all reads (including those mapping to multiple locations) is most appropriate for our purpose. However, we repeated our analysis using only uniquely mapping reads, to confirm that our results are robust to uncertainly in alignments (supplementary fig. S8*B*, Supplementary Material online).

**Fig. 2.**
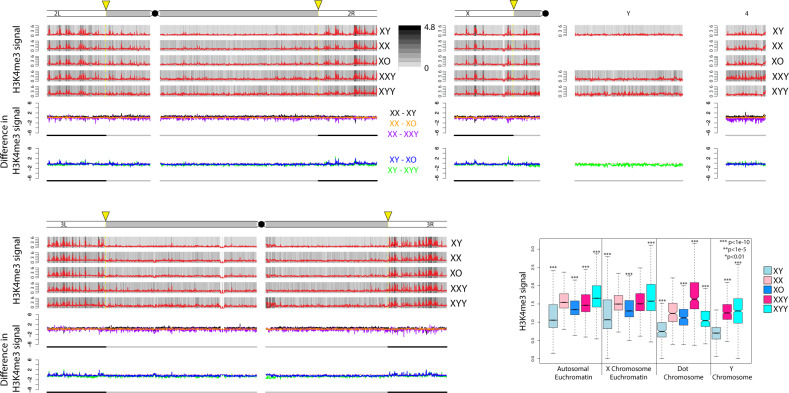
Enrichment of H3K4me3 for *Drosophila melanogaster* strains with different karyotypes across the euchromatin/heterochromatin boundary along each chromosome arm. We show the centromere-proximal 1-Mb euchromatic region of chromosomes 2, 3, and X, as well as the complete assembled heterochromatin region for each chromosome. For each karyotype, the enrichment in 5-kb windows is shown in red lines (normalized ratio of ChIP to input, see Materials and Methods), and the same data are shown in gray scale according to the scale in the upper right. Note that the enrichment profiles for all five karyotypes are plotted on the same scale to allow for direct comparisons. Below the enrichment profiles for each chromosome arm, subtraction plots show the absolute difference in signal of 5-kb windows between pairs of karyotypes along the chromosome arms. The cytogenomically defined heterochromatin is marked by gray bars and the euchromatin/heterochromatin boundary is indicated by a yellow arrow. The box plots show the ChIP signal for all 5-kb windows in different chromosomal regions, with boxes extending from the first to the third quartile and whiskers to the most extreme data point within 1.5 times the interquantile range. *P* values were calculated relative to XX females for XY males and XXY females and relative to XY males for X0 and XYY males; *P* values for the Y chromosome were calculated relative to XY males (Wilcoxon test). For genome-wide enrichment plots, see supplementary figure S13, Supplementary Material online.

**Fig. 3.**
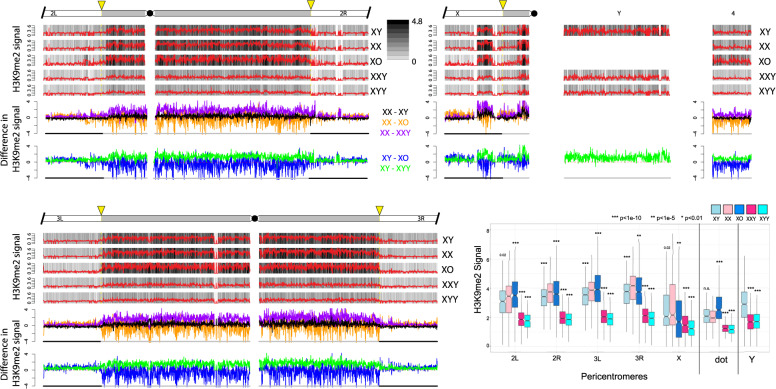
Enrichment of H3K9me2 for *Drosophila melanogaster* strains with different karyotypes across the euchromatin/heterochromatin boundary along each chromosome arm. We show the centromere-proximal 1-Mb euchromatic region of chromosomes 2, 3, and X, as well as the complete assembled heterochromatin region for each chromosome. For each karyotype, the enrichment in 5-kb windows is shown in red lines (normalized ratio of ChIP to input, see Materials and Methods), and the same data are shown in gray scale according to the scale in the upper right. Note that the enrichment profiles for all five karyotypes are plotted on the same scale to allow for direct comparisons. Below the enrichment profiles for each chromosome arm, subtraction plots show the absolute difference in signal of 5-kb windows between pairs of karyotypes along the chromosome arms. The cytogenomically defined heterochromatin is marked by gray bars and the euchromatin/heterochromatin boundary is indicated by a yellow arrow. The box plots show the ChIP signal for all 5-kb windows in different chromosomal regions, with boxes extending from the first to the third quartile and whiskers to the most extreme data point within 1.5 times the interquantile range. *P* values were calculated relative to XX females for XY males and XXY females and relative to XY males for X0 and XYY males; *P* values for the Y chromosome were calculated relative to XY males (Wilcoxon test).

**Fig. 4.**
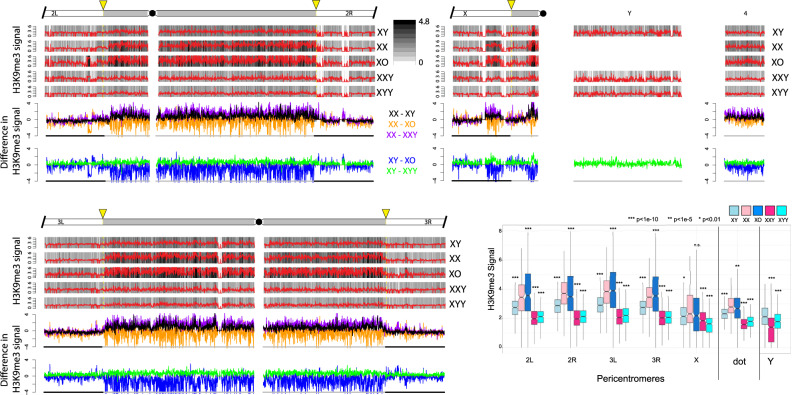
Enrichment of H3K9me3 for *Drosophila melanogaster* strains with different karyotypes across the euchromatin/heterochromatin boundary along each chromosome arm. These plots were made in the same manner as those for H3K9me2 (see fig. 3).

Signal for H3K4me3 is highly correlated across samples (supplementary table S6, Supplementary Material online), showing that our ChIP data are of high quality. In addition, H3K9me2 and H3K9me3 are known to have very similar genomic distributions (modENCODE Consortium et al. 2010), and they correlate well with each other for all samples (supplementary table S6, Supplementary Material online), and also with independent biological replicate ChIP data without a *D. miranda* chromatin spike (supplementary table S6 and figs. S9 and S10, Supplementary Material online; see Materials and Methods for details). Finally, we also generated replicate ChIP-seq data for H3K9me3 from X0, XXY, and XYY individuals using a different attached X stock, 4248 (supplementary fig. S11, Supplementary Material online). Again, these data are highly correlated and show similar genomic distributions and overall differences among the sex chromosome karyotypes as obtained for the 2549 strain (see below). Thus, our ChIP data are of high quality, and our results are reproducible using different mapping and normalization strategies, and across different histone modifications, independent biological replicates, and different genotypes. We used the total normalized number of *D. melanogaster* reads to compare the genome-wide distribution of chromatin modifications in flies with different sex chromosome karyotypes. Figure 2 shows the genomic distribution of the active H3K4me3 chromatin mark for the various karyotypes, and figures 3 and 4 show genomic distributions for the repressive H3K9me2/3 marks, respectively, at heterochromatic regions, and across the heterochromatin/euchromatin boundary (i.e., the transition of pericentromeric heterochromatin to euchromatin); for genome-wide enrichment plots, see supplementary figures S13–S15, Supplementary Material online.

### The Genomic Distribution of Active Chromatin Is Similar in Flies with Different Karyotypes

The histone modification H3K4me3 primarily associates with active genes (Guenther et al. 2007; Kharchenko et al. 2011) and is highly underrepresented in repeat-rich regions, including the Y chromosome; we thus expect that its relative abundance and genomic distribution is little influenced by the dose of Y chromosomes. Indeed, we find that H3K4me3 peaks are primarily located along the euchromatic chromosome arms, and highly deficient in pericentromeric regions, and along the Y chromosome (supplementary fig. S13, Supplementary Material online; for zoom-in at the heterochromatin/euchromatin boundary, see fig. 2). Genomic enrichment patterns of H3K4me3 are similar across sexes and flies with varying numbers of Y chromosomes (fig. 2 and supplementary figs. S9 and S13, Supplementary Material online), both when comparing the relative position of peaks, but also the absolute magnitude of signal across samples (fig. 2 and supplementary figs. S9 and S13, Supplementary Material online). This confirms our expectation that Y dose should not dramatically influence the distribution of active chromatin marks and also suggests that our normalization procedure is accurate in quantifying relative abundance of histone modifications across samples. Western blots confirm our inferences based on ChIP-seq, that is, that H3K4me3 signal is similar across flies with different karyotypes (supplementary fig. S12, Supplementary Material online).

### Heterochromatic Histone Modifications in Wildtype Flies

We investigated the genomic distribution of two histone marks that are associated with heterochromatin formation, H3K9me2 and H3K9me3 (Kharchenko et al. 2011). If the Y chromosome indeed acts as a sink for components of the heterochromatin machinery, we expect global differences in the enrichment patterns of heterochromatic histone modifications across strains with different numbers of Y chromosomes, or more generally, across flies with different amounts of repetitive DNA (see fig. 1*A* and *B*). Specifically, we expect that as the repeat content increases, flies should harbor less H3K9me2/3 at their heterochromatic regions. In wildtype Drosophila, heterochromatin is highly enriched in pericentromeric regions, the small dot chromosome, and along the entire length of the Y chromosome (Hannah 1951; Gatti and Pimpinelli 1983; Bonaccorsi and Lohe 1991). Note that the *D. melanogaster* Y chromosome is estimated to be about 40 Mb (i.e., 20% of the haploid male genome; Gatti and Pimpinelli 1983; Hoskins et al. 2002), but only 3.7 Mb (i.e., <10%) of the Y chromosome has been assembled. Similarly, other heterochromatic regions are also only partly assembled: 1.5 Mb (∼25% of the pericentromeric heterochromatin) on chromosome 2L, 5.4 Mb (∼50%) on 2R, 5.1 Mb (∼50%) on 3L, 4.2 Mb (∼50%) on 3R, and only 0.9 Mb (i.e., only about 5% of the pericentromeric heterochromatin) on the X chromosome. Thus, our genome mapping analysis will underestimate the extent of heterochromatic histone modifications that are associated with the Y chromosome and other repetitive regions. Also note that the pericentromeric heterochromatin along the X chromosome is noncontinuous (figs. 2 and 3; see also Riddle et al. 2011), with the more distal heterochromatic block encompassing the *flamenco* locus (Goriaux et al. 2014).

**Fig. 5.**
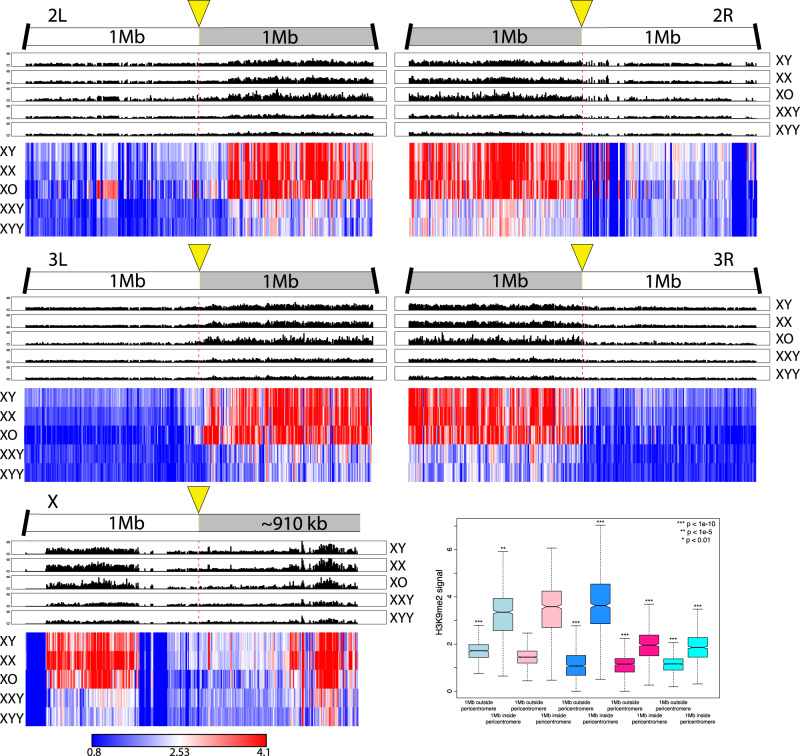
Enrichment of H3K9me2 within 1 Mb of the heterochromatin/euchromatin boundaries (as defined in the Release 6 of the *Drosophila melanogaster* genome [Hoskins et al. 2015]). The upper panels show H3K9me2 signal in 5-kb windows for each chromosome arm, and the bottom panel shows scaled heatmaps for the same 5-kb windows, to allow direct comparisons of H3K9me2 signal across samples. For H3K9me3 plots, see supplementary figure S16, Supplementary Material online. Box plots show H3K9me2 signal of 5-kb windows in euchromatic regions 1 Mb outside the pericentromere versus 1 Mb inside the heterochromatin/euchromatin boundary. Significance values are all calculated using the Wilcoxon test.

Overall, we find that levels of heterochromatin enrichment are similar for the H3K9me2 and H3K9me3 marks but differ between flies with varying amount of repetitive DNA (figs. 3 and 4; for genome-wide plots, see supplementary figs. S14 and S15, Supplementary Material online). The male-specific Y chromosome is highly enriched for both of these repressive histone modifications in wildtype males, and we find that wildtype females have slightly higher levels of H3K9me2/3 enrichment than males in their pericentromeric regions, and on the dot chromosome, relative to euchromatic background levels (figs. 3 and 4). Moreover, the heterochromatin/euchromatin boundary is slightly less clearly discernable from H3K9me2/3 enrichment patterns for males relative to females (fig. 5 and supplementary figs. S16 and S17, Supplementary Material online). Western blots suggest that males harbor slightly more H3K9me2/3 compared with females (supplementary fig. S12, Supplementary Material online). Thus, we find strong enrichment of the heterochromatic histone modifications on the Y and their relative deficiency at pericentromeric regions on autosomes and the X in wildtype males relative to females, despite similar amounts of overall H3K9me2/3. This observation is consistent with the hypothesis that the repeat-rich Y chromosome acts as a sink for components of the heterochromatic machinery, resulting in a relative paucity of heterochromatic histone modifications elsewhere in the genome. However, despite quantitative differences in levels of heterochromatic histone modifications, overall patterns of H3K9me2/3 enrichment are similar between sexes.

### Heterochromatic Histone Modifications in X0, XXY, and XYY Flies

To investigate the Y chromosome’s role in the genome-wide distribution and enrichment for heterochromatic components, we studied histone modification profiles from female flies containing a Y chromosome (XXY females), and males with either zero or two Y chromosomes (X0 vs. XYY males). Female Drosophila that contain a wildtype Y chromosome show clear enrichment for both heterochromatic histone modifications on the Y chromosome, but an overall reduction in levels of H3K9me2/3 relative to wildtype females, both at pericentromeric regions and along the dot (figs. 3 and 4). The genomic distribution of H3K9me2/3 in XXY females is consistent with the model of the Y chromosome acting as a sink for components of the heterochromatin machinery, sequestering heterochromatic proteins to the Y chromosome and diluting them away from autosomal and X-linked targets. XXY females also show less heterochromatic histone modifications at pericentromeric regions and the dot relative to wildtype XY males (figs. 3 and 4). This is consistent with the higher repeat content in XXY flies compared with XY flies—due to the large heterochromatic block on the X—contributing to the heterochromatin sink effect. This suggests that the effect of the Y chromosome on heterochromatin distribution is not a unique property of the Y but instead a result of a large amount of any additional repetitive sequence. XYY males harbor the highest amount of repetitive DNA and show severely decreased levels of H3K9me2/3 enrichment along repeat-rich, normally heterochromatic regions, including their Y chromosomes, pericentromeric regions, and along the dot, relative to levels found in other karyotypes investigated (figs. 3 and 4).

X0 males, on the other hand, have the lowest repeat content of all flies and show the strongest enrichment of heterochromatic histone modifications at pericentromeric regions and along the dot chromosome (figs. 3 and 4). Enrichment levels of H3K9me2/3 at repetitive regions (pericentromere and the dot) relative to euchromatic background levels in X0 males are well above that of wildtype males and also wildtype females (or XXY females, which have the same autosomal background as X0 flies; figs. 3 and 4). Similar patterns of redistribution of heterochromatin are observed in a biological replicate without a spike (supplementary fig. S10, Supplementary Material online), and in X0, XXY, and XYY flies that were generated using a different attached-X/XY stock, 4248 (supplementary fig. S11, Supplementary Material online), demonstrating that our findings are robust in different genetic backgrounds. Together, our data provide evidence that Y chromosomes, and repetitive DNA in general, affect heterochromatin formation genome-wide, consistent with a model of the Y chromosome or other large blocks of repetitive sequences acting as heterochromatin sinks, possibly by redistributing heterochromatin components across the genome. The sink effect of additional heterochromatin is roughly positively correlated with increasing amounts of repetitive DNA: We typically see increasingly less heterochromatin form at the pericentromeres and the dot chromosome as the repeat content increases (see boxplot in figs. 3 and 4). Note, however, that some karyotypes or pericentromeric regions do not always follow this overall pattern (figs. 3 and 4). In particular, XX females and X0 males typically have the highest amount of heterochromatin at their pericentromeres across chromosomes, but their rank often differs: pericentromere 2L, for example, has higher levels of both H3K9me2/3 in X0 flies, but 2R shows higher enrichment in XX females. Wildtype XY males typically show lower enrichment levels for H3K9me2/3 at all chromosomes relative to X0 and XX individuals. Heterochromatin is noticeably reduced in XXY and XYY individuals across the genome compared with other karyotypes, but their relative rank also differs among marks and chromosomes, with H3K9me2 typically being higher in XXY flies, but H3K9me3 often being slightly less. Whether this variation reflects noise in our methodology or underlying biological differences due to heterogeneity in the repeat content of heterochromatic regions across chromosomes is unclear.

The depletion of heterochromatic histone modifications from pericentromeric regions causes the euchromatin/heterochromatin boundaries to be significantly diluted in XXY and XYY individuals (fig. 5 and supplementary figs. S16 and S17, Supplementary Material online). X0 males, in contrast, show spreading of their pericentromeric heterochromatin into chromosome arms that are normally euchromatic in wildtype flies, which is consistent with previous studies that found enhanced PEV in X0 males (fig. 5 and supplementary figs. S16 and S17, Supplementary Material online; Belyaeva et al. 1993; Wallrath and Elgin 1995).

Overall, we see that increasing the amount of repetitive DNA by changing the dose of both sex chromosomes corresponds with a decrease in the signal of heterochromatic histone modifications at pericentromeric regions and along the dot chromosome. This is consistent with a model of stoichiometric balance between protein components involved in the formation of heterochromatin and the amount of repetitive DNA sequences within a genome. Together, ChIP-seq profiles of histone modifications in wildtype flies, X0 and XYY males, and XXY females, support the hypothesis that the Y chromosome acts as a heterochromatin sink in Drosophila.

### Sex Chromosome Dose and Gene Expression

**Fig. 6.**
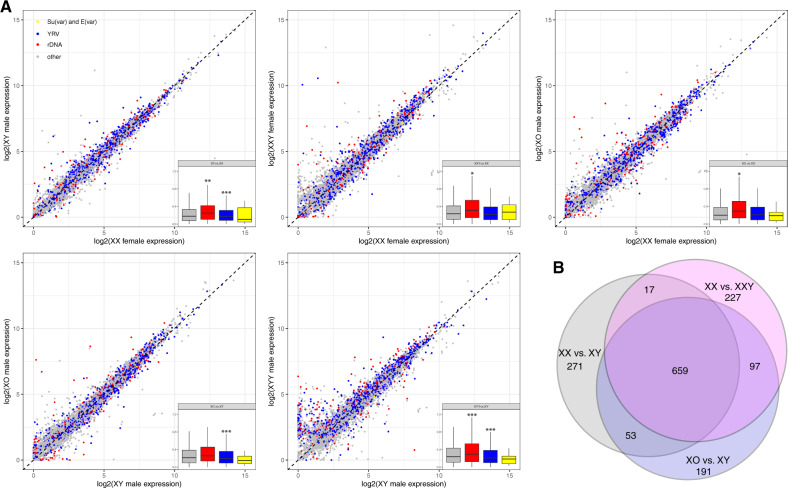
Gene expression variation between flies with different sex chromosome karyotypes. (*A*) Pairwise expression comparisons for flies with different karyotypes. Genes marked in red are susceptible to YRV (Sackton and Hartl 2013), genes in blue are sensitive to rDNA copy number (Paredes et al. 2011), and genes in yellow are genetically defined Su(var) and E(var) genes in *Drosophila melanogaster* (Elgin and Reuter 2013); gray genes are all other genes. (*B*) Overlap of top 1,000 differentially expressed genes between wildtype XY male and XX female, and males and females with and without Y chromosomes, that is, XX versus XXY females and XY versus X0 males.

Polymorphic Y chromosomes affect expression of hundreds of autosomal and X-linked genes in *D. melanogaster*, a phenomenon known as Y-linked regulatory variation (YRV) (Dimitri and Pisano 1989; Lemos et al. 2008, 2010; Sackton et al. 2011). To test if genes that respond to YRV are also expressed differentially in flies with different sex chromosome configurations, we collected replicate RNA-seq data from heads for wildtype males and females, as well as X0, XXY, and XYY flies (see supplementary table S7, Supplementary Material online, for overview of RNA-seq data, and supplementary appendix S1, Supplementary Material online, for summary of expression values). As noted above, protein-coding Y-linked genes in Drosophila are only expressed in male germ line and thus cannot directly contribute to differences in expression profiles in head samples among flies with different numbers of Y chromosomes. Overall, we find that hundreds of genes show differential expression among flies with different sex chromosome karyotypes (fig. 6*A*). Gene ontology (GO) analysis revealed that differentially expressed genes tend to be enriched for functions associated with reproductive processes (supplementary table S8, Supplementary Material online) and are not simply clustered around pericentromeric regions (supplementary figs. S18 and S19, Supplementary Material online). Genes that are expressed most differently between X0 and XY males, and XX and XXY females, show significantly greater difference in H3K9me2 signal compared with all genes, whereas these genes have significantly less difference in H3K4me3 signal compared with all genes (supplementary fig. S20, Supplementary Material online). This is consistent with the hypothesis that the Y chromosome redistributes heterochromatin components and can thereby influence the expression of hundreds of genes. However, we see no global relationship between gene expression differences and H3K9me2/3 enrichment levels across all genes (supplementary figs. S21 and S22, Supplementary Material online). This suggests that the effect of the Y chromosome on heterochromatin and its effect on gene expression are not explained by a simple model whereby the Y chromosome modifies heterochromatin formation and thereby directly modifies gene expression across the entire genome. Indeed, the majority of genes are not targeted by H3K9me2/3 above background levels in any of the karyotypes investigated, and thus those marks are unlikely to directly influence global gene expression patterns.

We used a consensus set of 678 genes that were classified as susceptible to YRV (Sackton and Hartl 2013) and found that these genes were generally expressed more differently between different sex chromosome karyotypes compared with random genes (fig. 6*A*). This suggests that a similar mechanism is underlying both YRV and gene expression differences in flies with different sex chromosome configurations. Similarly, we find that genes whose expression is sensitive to rDNA copy number (Paredes et al. 2011) tend to be differentially expressed between karyotypes with different number of Y chromosomes; however, not all rDNA-sensitive genes show differential expression between our karyotypes, suggesting that the Y-linked rDNA arrays are not the primary driver of differential gene expression in our study (fig. 6*A*). Genes that are genetically defined to either suppress or enhance silencing in assays for PEV in *D. melanogaster*, that is, Su(var) and E(var) genes (Elgin and Reuter 2013), are expressed at similar levels in flies with different karyotypes (fig. 6*A*). This is consistent with our Western blots that reveal no consistent differences in total H3K9me2/3 levels among flies with different sex chromosome configurations (supplementary fig. S12, Supplementary Material online).

Interestingly, genes susceptible to YRV are more likely to be differentially expressed between wildtype sexes, and genes that are differentially expressed between males and females in head tissue tend to also be differentially expressed between X0 and XY males, or XX and XXY females (*P* < 1e-6, permutation test, fig. 6*B* and supplementary fig. S23, Supplementary Material online). In particular, 659 of the top 1,000 genes that are differentially expressed between wildtype XX females and XY males, versus X0 and XY males versus XX and XXY females overlap, whereas we only expect 11 by chance. This suggests that a substantial fraction of sex-biased expression in somatic tissues may simply be an indirect consequence of the absence or presence of the Y, that is, the sink effect of the Y chromosome may contribute to sex-biased expression patterns in *D. melanogaster*.

### Repeat Reactivation in XXY and XYY Flies

**Fig. 7.**
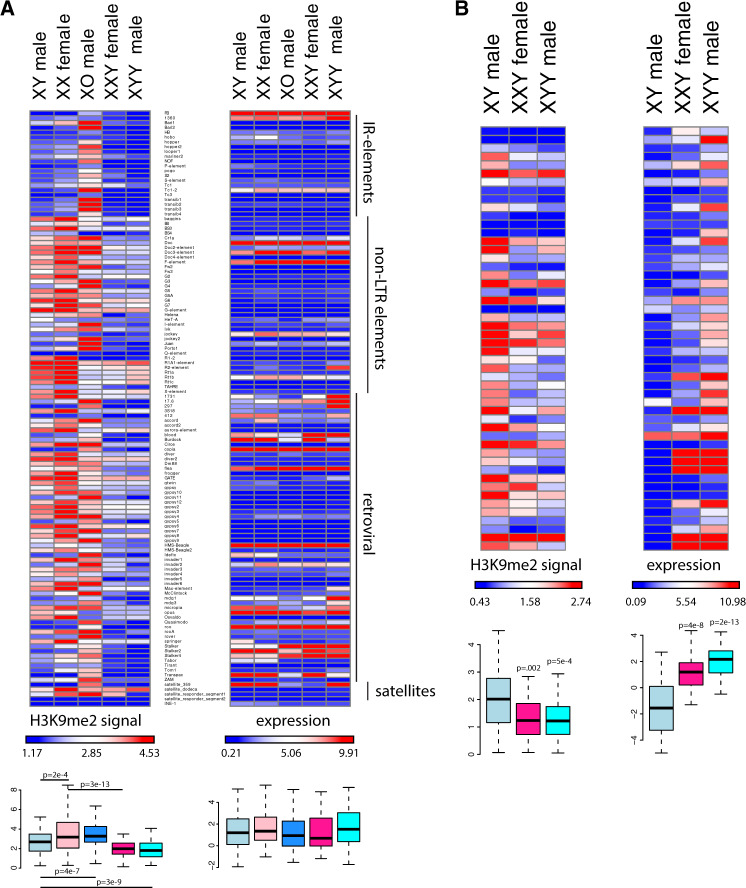
Chromatin and expression patterns at TE families. Shown is enrichment of H3K9me2 at different TE families (relative to genome-wide levels) and their expression levels for the various karyotypes. For H3K9me3 plots, see supplementary figure S24, Supplementary Material online. (*A*) All repeats from the library of consensus TEs and satellites from FlyBase. Boxplots summarize H3K9me2 and expression values across all repeats, and significance values were calculated using the Wilcoxon test; all comparisons of expression levels were not significant. (*B*) Putatively Y-linked (male-specific) de novo assembled repeats only. Boxplots summarize H3K9me2 and expression values across all putatively Y-linked repeats, and *P* values were calculated relative to XY males using the Wilcoxon test.

Heterochromatin is established during early embryogenesis and leads to the transcriptional silencing of repetitive DNA and TEs (Elgin and Reuter 2013). We used our RNA-seq data to assess whether changes in chromatin structure due to Y chromosome dose are associated with changes in gene expression patterns of repetitive elements. We first used consensus sequences of known TEs annotated by FlyBase (flybase.org) and found that overall repeat content correlated negatively with H3K9me2/3 enrichment at TEs: X0 flies had the highest level of H3K9me2/3 enrichment across TE families, followed by XX and XY wildtype flies, and XXY and XYY flies having the lowest amount of heterochromatin marks at their TEs (*P* < 0.01 for each comparison; fig. 7*A* and supplementary fig. S24*A*, Supplementary Material online; note that these estimates are corrected for differences in copy numbers between repeats, by looking at the enrichment of H3K9me2/3 enrichment over input for each karyotype). Despite dramatic differences in overall levels of repressive histone marks across repeat families, levels of expression for the various TEs between karyotypes are very similar (*P* > 0.05, fig. 7*A* and supplementary fig. S25, Supplementary Material online). A subset of TEs shows an increase in expression in XYY males compared with other samples, including at least five retroviral elements (1,731, 297, Max element, mdg1, and mdg3, supplementary fig. S25, Supplementary Material online). Increased expression of these repeats appears in part be driven by an increased copy number in the XYY male genome; if we correct for genomic copy number, we find that only three of these repeats (1,731, 297, and Max element) are expressed more highly in XYY males compared with the other karyotypes (supplementary fig. S26, Supplementary Material online). Thus, despite global differences in heterochromatin formation associated with repeats across karyotypes, this does not manifest itself in a global de-repression of TEs but seems to instead involve de-repression of just a subset of TE families. Note that a loss of heterochromatin at pericentromeric regions and the Y chromosome should not necessarily result in increased expression across all TE families present. On one hand, most TEs located in the pericentromere and the Y chromosome are partial and nonfunctional copies that have lost their ability to transpose (Ananiev et al. 1984; Pimpinelli et al. 1995). In addition, we assayed gene expression in somatic head tissue, and many TE families only mobilize in the germline (Charlesworth and Langley 1989).

Most of the Y chromosome has not yet been assembled (Hoskins et al. 2015), including its repetitive elements, and we were interested in whether expression of Y-linked repeats would be particularly sensitive to Y chromosome dosage. We thus used a de novo approach to identify male-specific Y-linked repeats that does not rely on a genome assembly, but instead uses kmer abundances from next generation sequencing reads to produce a repeat library (Koch et al. 2014). We then mapped male and female genomic reads from the Canton-S strain back to our de novo assembled repeat library, in order to infer Y-linkage for repeats that were only covered by male genomic reads (supplementary figs. S27 and S28, Supplementary Material online; see supplementary appendix S2, Supplementary Material online, for a list of male-specific repeats). Male-specific repeats are highly enriched for H3K9me2/3 in wildtype males and transcriptionally silenced (fig. 7*B*). However, although Y-linked repeats show similar enrichment for the H3K9me3 mark in all karyotypes (supplementary fig. S24*B*, Supplementary Material online), XXY females and XYY males are highly deficient for H3K9me2 at Y-linked repeats, and expression of Y-linked repeats is de-repressed relative to wildtype males (fig. 7*B* and supplementary fig. S29, Supplementary Material online). If we account for differences in copy number of the Y-linked repeats, we still find that Y-linked repeats are expressed more highly in XXY females and XYY males compared with wildtype males (supplementary fig. S30, Supplementary Material online). Thus, consistent with the ChIP-seq data that showed low levels of heterochromatic histone modifications (especially H3K9me2) along the Y of XXY females or the two Y chromosomes of XYY males, relative to wildtype males, our gene expression data demonstrate that Y-linked repeats become transcriptionally activated in female flies that normally do not have a Y chromosome, or male flies with double the dose of Y-linked repeats, and this is not simply a consequence of an increased copy number of Y-linked repeats.

## Discussion

### Dosage Effects of Chromatin Components and Repetitive DNA

Many eukaryotic genomes contain large amounts of selfish, repetitive DNA; in the Drosophila strains investigated, for example, the heterochromatin content varies from about 1/3 to 1/2 of the genome (see fig. 1*B*). Transcriptional silencing of repeats through heterochromatin formation is one way to alleviate the deleterious effects of repetitive DNA (Elgin and Reuter 2013). Studies of PEV in *D. melanogaster* have yielded important insights into the biology of heterochromatin (Muller 1930; Schultz 1936; Zhimulev et al. 1986) and frequently found dose-dependent effects of chromatin proteins and trans-activating factors (Weiler and Wakimoto 1995). For example, depletion of HP1, an important protein involved in both the recruitment and maintenance of heterochromatic histone modifications, suppresses variegation (i.e., it results in less heterochromatin formation and thus less suppression at a reporter gene; Eissenberg et al. 1990), whereas increased dosage of HP1 enhances variegation (i.e., it increases silencing through increased heterochromatin formation; Eissenberg et al. 1992). Addition of extra heterochromatin to the genome suppresses variegation, whereas its subtraction enhances variegated gene expression (Baker 1968; Spofford 1976; Gatti and Pimpinelli 1992). In *D. melanogaster*, the Y chromosome is a potent suppressor of variegation, that is, it induces less heterochromatin at a reporter gene (Gowen and Gay 1934), and *D. melanogaster* males with different Y chromosomes in otherwise identical genetic backgrounds vary in their propensity to silence a heterochromatin-sensitive reporter gene in PEV assays (Lemos et al. 2010).

Highly repetitive Y chromosomes are thought to sequester heterochromatic factors that are present in only limited amounts (Dimitri and Pisano 1989), and different Y chromosomes vary in their repeat content and thus the extent to which they sequester those heterochromatin components, thereby influencing PEV. Reporter gene assays, however, do not directly probe the integrity or amount of heterochromatin at repetitive regions that exert PEV through spreading of heterochromatin, nor do they directly demonstrate changes in the spreading of heterochromatin along the chromosome (Spofford 1976; Dimitri and Pisano 1989; Gatti and Pimpinelli 1992). Under the heterochromatin sink model, changes in the amount of repetitive DNA should globally modify the amount of heterochromatin formed at repeats. Specifically, decreasing the amount of repetitive DNA should result in increased levels of heterochromatin at repetitive regions, since heterochromatic factors that are present in only limited amounts can be sequestered at the existing repeats at higher concentration, whereas increased amounts of repeats would result in the opposite effect.

In our study, we directly demonstrate that the Y chromosome, and repeat-rich DNA in general, can act to globally affect heterochromatin formation in *D. melanogaster*. Consistent with the heterochromatin sink model, we find that increasing the amount of repetitive DNA generally decreases the amount of H3K9me2/3 enrichment at repeat-rich regions, such as pericentromeres, the dot, or the Y chromosome. Individuals with the lowest repeat content (X0 males in our experiment) often show the highest enrichment of H3K9me2/3 in repeat-rich regions, and the pericentromeric heterochromatin on the autosomes of X0 flies clearly extends into genomic regions that are normally euchromatic in wildtype *D. melanogaster*. Wildtype females show slightly higher H3K9me2/3 levels at their pericentromeric regions and the dot chromosome and a slightly sharper euchromatin/heterochromatin boundary at autosomes compared with wildtype males. Indeed, females generally show a higher degree of silencing in assays for PEV, suggesting that normally euchromatic regions are more prone to acquire a heterochromatic conformation in females (Wallrath and Elgin 1995; Girton and Johansen 2008).

XYY males and XXY females, on the other hand, show a dramatic reduction of H3K9me2/3 enrichment at repeat-rich regions, and the boundaries between the heterochromatic pericentromere and the euchromatic chromosome arms become blurry. Overall, the sink effect of additional heterochromatin appears proportional to increasing amounts of repetitive DNA, consistent with studies based on PEV (Dimitri and Pisano 1989). Thus, this dosage sensitivity of H3K9me2/3 enrichment in repetitive regions suggests that there is a stoichiometric balance among protein components and total repeat content of the genome to maintain proper heterochromatic silencing. Redistribution of silencing marks in flies with higher repeat content suggests limited buffering in heterochromatin formation. Increased repeat content within a genome could in principle be compensated for by increasing the quantity of heterochromatin factors that might be limited within a cell, in order to ensure chromatin homeostasis. Reduced heterochromatin formation in XXY and XYY flies suggests that mechanisms to maintain heterochromatin homeostasis, if present, are limited, and cannot compensate for extra repetitive DNA found in these flies. This is consistent with our Western blots that reveal no consistent differences in H3K9me2/3 levels among flies with different sex chromosome configurations (supplementary fig. S12, Supplementary Material online), and PEV assays demonstrating that addition of extra repetitive DNA to the genome suppresses variegation, whereas its subtraction enhances variegated gene expression (Baker 1968; Spofford 1976; Gatti and Pimpinelli 1992).

### Functional Heterogeneity of Heterochromatin

Most DNA sequences that comprise the various heterochromatic elements are not unique and specific to chromosomes or chromosomal segments but are shared with other genomic regions; most satellite DNA repeats map to multiple genomic sites (Bonaccorsi and Lohe 1991), and so do nearly all the TEs (Pimpinelli et al. 1995). All highly repetitive blocks in the *D. melanogaster* genome, including both arms of the Y chromosome, the heterochromatic segments located at the base of the X chromosome and the left and right arms of chromosomes 2 and 3, and the fourth chromosome heterochromatin are all effective in inducing PEV (Spofford 1976). An important open question is whether the ability to suppress variegation is a general property of all heterochromatic regions, or whether it can be ascribed to specific heterochromatic sites. PEV suppression exerted by the Y chromosome was mapped using a variety of cytologically determined Y-chromosomal deficiencies and Y-linked fragments (Dimitri and Pisano 1989). The suppression effect exerted by the Y chromosome was found to be positively related to the size of the Y-derived DNA and was not attributable to any discrete Y region; Y chromosomes that were cytologically different yet retain similar amounts of heterochromatin were found to be equally effective suppressors (Dimitri and Pisano 1989). Thus, at the level of resolution provided by these cytogenetic studies, all Y fragments appear to be similarly effective in influencing global chromatin structure, as assayed by PEV assays. In our study, we also find that there is an inverse relationship between the amount of repetitive DNA present, and the amount of heterochromatin induced at repeats, by varying the repeats derived from both the X and the Y chromosome. This is consistent with the notion that there is limited heterogeneity among repeats in influencing global chromatin structure. Despite these global trends of heterochromatin redistribution among karyotypes with varying repeat content, variation in relative heterochromatin enrichment exists across karyotypes and among genomic regions that does not follow this simple linear relationship (see figs. 3 and 4). Whether this variation reflects experimental noise or biological differences in heterochromatin formation due to heterogeneity in underlying repeat sequence composition is unclear. It will be of great interest to more carefully characterize the effects of specific repeat elements on the Y chromosome, such as the rDNA cluster, or specific types of satellites, to directly address the question of how uniform the sink effect is across different repeat types.

### Functional Consequences of the Y Chromosome’s Global Effects on Heterochromatin

Analyses of gene expression profiles suggest that global changes in heterochromatic histone modifications can have broad functional consequences for the organism. Specifically, we show that hundreds of genes are differentially expressed in individuals that differ in their sex chromosome karyotype, and genes that are susceptible to YRV are more prone to be differentially expressed in individuals with different sex chromosome complements.

We find that increasing the amount of repetitive DNA leads to a decrease in heterochromatic histone modification signal at TEs. XYY males and XXY females have low levels of H3K9me2/3 signal in TEs, and especially so in male-specific repeats, and we show that this deficiency of heterochromatin is associated with a de-repression of Y-linked repeats that we detect as an increase in expression levels of these repeats. Thus, although fruit flies have efficient mechanisms in place to silence wildtype levels of repetitive DNA, a large increase in the amount of repetitive sequences, caused by introducing additional Y chromosomes, limits the organism’s ability to form heterochromatin and those additional repeats apparently cannot be efficiently silenced.

Although our study was aimed at testing the role of a heterochromatin sink driving transcriptional regulation, other factors might contribute to the observed expression variation among strains (see Francisco and Lemos 2014 for a detailed discussion). In particular, the rDNA cluster is present on both the X and the Y chromosome, and variation in rDNA numbers has been shown to explain ∼20–40% of YRV in *D. melanogaster* (Paredes et al. 2011). Additionally, heterochromatic regions, including the Y, produce abundant piRNAs and possibly endo-siRNA, which could modulate expression of a large number of genes or TEs (Brennecke et al. 2007; Malone and Hannon 2009). Furthermore, hundreds of genes are differentially expressed between adult male *D. melanogaster* that differ in the maternal and paternal origin of the sex chromosomes (Lemos et al. 2014), and genomic imprinting may contribute to observed expression differences among strains. Detailed molecular studies will be necessary to characterize the mechanistic basis of expression variation among flies with different numbers of sex chromosomes and amounts of heterochromatin.

### Heterochromatin/Euchromatin Balance between Sexes

Males contain a Y chromosome that is highly repetitive and heterochromatic, and which may shift the genome-wide heterochromatin/euchromatin balance between the sexes (Brown and Bachtrog 2014). In particular, if the Y chromosome sequesters proteins required for heterochromatin formation, males may be more sensitive to perturbations of the balance between repetitive sequence content and heterochromatic protein components and might have lower levels of heterochromatin-like features in the rest of their genome, as compared with females (Brown and Bachtrog 2014). Indeed, RNAi knockdown of the heterochromatin protein HP1 preferentially reduces male viability (Liu et al. 2005), and the presence of Y-linked heterochromatin is thought to underlie this differential sensitivity. Heterochromatin formation is temperature sensitive, and female Drosophila are more tolerant of heat shock, survive heat-induced knockdown better (Yamamoto and Ohba 1982), and become sterile at higher temperatures than males (David et al. 2005), and it is possible that differences in the chromatin landscape may contribute to sex-specific differences in heat stress response. Indeed, the Y chromosome is responsible for much of the genetic variation of heat-induced male sterility found across populations (Rohmer et al. 2004). Also, as mentioned, female flies show stronger silencing in assays for PEV (Wallrath and Elgin 1995; Girton and Johansen 2008), consistent with having more heterochromatin protein components relative to repetitive sequences, which can then spread into reporter genes more readily.

Many recent studies in animals have shown that a large portion of the transcriptome in animals is sex biased (Ranz et al. 2003; Mank et al. 2008). Sex-biased expression patterns are typically seen as an adaptation to form the basis of sexually dimorphic phenotypes (Parsch and Ellegren 2013). In Drosophila, most sex-biased expression patterns are due to differences in expression in sex-specific tissues (i.e., gonads; Meisel 2011; Assis et al. 2012); however, hundreds of genes also show differential expression in shared, somatic tissues (Meisel 2011; Assis et al. 2012). Interestingly, we find that a similar set of genes that show differences in expression patterns between males and females (in head) are also differentially expressed between XY and X0 males, or XX and XXY females. This suggests that not sex per se, but the absence or presence of the Y chromosome is responsible for much of the differences in expression patterns between sexes. Sex-biased gene expression is normally interpreted as a sex-specific adaptation to optimize expression levels of genes in males and females (Parsch and Ellegren 2013). However, our results suggest that it is also possible that sex-biased expression patterns are simply an indirect consequence of global differences in the chromatin structure between males and females, due to the presence of a large repetitive Y chromosome in males. Thus, this would imply that sex-specific adaptations in gene expression are less common than often suggested. Contrasting species with differing amounts of sex-specific heterochromatin (i.e., sex chromosomes at different stages of differentiation) should help to resolve the importance of selection versus a chromatin sink in driving sex-biased gene expression patterns.

### Evolutionary Implications of a Heterochromatin Sink

Most eukaryotic genomes harbor large amounts of repetitive DNA, including TEs and satellite DNA (Hoskins et al. 2015), and repeats comprise a highly dynamic part of the genome. Closely related species often have nearly complete turnover of the types and abundances of satellite repeats (Kamm et al. 1995; Lohe and Roberts 2000; Wei et al. 2018), and repetitive DNA varies even among species within a population (Wei et al. 2014). Early cytogenetic studies have shown that individuals within a population can differ greatly in how much repetitive heterochromatic DNA they contain. The size of the pericentromeric heterochromatic block on the *D. melanogaster* X chromosome, for example, varies by about 2-fold among strains (i.e., between 10 and 20 Mb in size; Halfer 1981), and dramatic variation in size and morphology of the Y chromosome has been reported in natural populations of *D. pseudoobscura* (Dobzhansky 1937). Moreover, haploid genome size estimates of different *D. melanogaster* strains using flow cytometry differ by almost 100 Mb, and the vast majority of this variation is thought to result from differences in repetitive heterochromatin (Bosco et al. 2007). Similarly, a recent bioinformatics analysis that identified and quantified simple sequence repeats from whole genome sequences also found a 2.5-fold difference in their abundance between *D. melanogaster* strains (Wei et al. 2014). Thus, natural variation in repetitive DNA among individuals may in fact span a wider range than that across sex chromosome karyotypes investigated here. This implies that repetitive DNA might serve as an important determinant of global chromatin dynamics in natural populations and may be an important modifier of the differential expression of genes and TEs between individuals. Redistribution of heterochromatin due to differences in repeat content can thus have important consequences on individual fitness and phenotypic evolution. Indeed, the Y chromosome of *D. melanogaster* has been shown to effect male fitness (Chippindale and Rice 2001), temperature sensitivity of spermatogenesis (Rohmer et al. 2004), and life span (Griffin et al. 2015), despite having few protein-coding genes and a near absence of sequence polymorphism in Y-linked protein-coding genes. Thus, differences in repeat content between individuals, sexes, and species might play an important role in phenotypic evolution, by globally modulating gene expression via epigenetic mechanisms.

## Materials and Methods

### Drosophila Strains

Fly strains were obtained from the Bloomington Stock Center. The following strains were used: Canton-S, 2549 (C(1;Y),y^1^cv^1^v^1^B/0 & C(1)RM, y^1^v^1^/0), and 4248 (C(1)RM, y^1^pn^1^v^1^/C(1;Y),y^1^B^1^, y^1^B^1^/0; sv^spa-pol^). The crossing scheme used to obtain X0 and XYY males and XXY females is depicted in figure 1*B*. For chromatin and gene expression analyses, flies were grown in incubators at 25 °C, 32% of relative humidity, and 12 h light. Newly emerged adults were collected and aged for 8 days in mixed-sex vials under the same rearing condition before they were flash-frozen in liquid nitrogen and stored at −80 °C.

### Genome Size Estimation

We estimated genome size of the five karyotypes of interest using flow cytometry methods similar to those described in Ellis et al. (2014). Briefly, samples were prepared by using a 2-ml Dounce to homogenize one head each from an internal control (*Drosophila virilis* female, 1C = 328 Mb) and one of the five karyotypes in Galbraith buffer (44 mM magnesium chloride, 34 mM sodium citrate, 0.1% [v/v] Triton X-100, 20 mM 3-(N-morpholino)propanesulfonic acid, and 1 mg/ml RNAse I, pH 7.2). After homogenizing samples with 15–20 strokes, samples were filtered using a nylon mesh filter and incubated on ice for 45 min in 25 μg/ml propidium iodide. Using a BD Biosciences LSR II flow cytometer, we measured 10,000 cells for each unknown and internal control sample. We ran samples at 10–60 events per second at 473 V using a PE laser at 488 nm. Fluorescence for each *D. melanogaster* karyotype was measured using the FACSDiva 6.2 software and recorded as the mode of the sample’s fluorescent peak interval. We calculated the genome size of the five karyotypes by multiplying the known genome size of *D. virilis* (328 Mb) by the ratio of the propidium iodide fluorescence in the unknown karyotype to the *D. virilis* control.

### Repeat Estimation from Genomic Reads

We also performed an independent characterization of repetitive elements using de novo assembly of repeats with dnaPipeTE (Goubert et al. 2015). We extracted genomic DNA from wildtype Canton-S male and females with the abdomens removed and performed a standard phenol–chloroform extraction. Raw reads were filtered for bacteria and mitochondrial DNA. We ran dnaPipeTE on all filtered reads using the same parameters: -genome_size 175000000 –genome_coverage 0.50 -sample_number 2. An overview of all genomic data generated can be found in supplementary table S2, Supplementary Material online.

### Western Blotting

We performed Western blots from acid-extracted histones, probing for H3K9me2, H3K9me3, H3K4me3, and total H3. Briefly, ∼30 flies of each karyotype were dissected on dry ice to remove the abdomen. The resulting heads and thoraces were ground in Phosphate-buffered saline (PBS) plus 10 mM sodium butyrate and were acid extracted overnight at 4 °C. Samples were then run on a 4–12% gradient bis-tris gel and transferred to a nitrocellulose membrane using Invitrogen’s iBlot Dry Transfer Device. After blocking with 5% milk in PBS, we incubated membranes overnight with either 1:1,000 H3K9me2 antibody (Abcam ab1220), 1:2,000 H3K9me3 antibody (Abcam ab8898), 1:2,000 H3K4me3 antibody (Abcam ab8580), or 1:2,000 H3 antibody (Abcam ab1791) in Hikari Signal Enhancer (Nacalai 02272). We then incubated membranes with 1:2,500 secondary antibody (Licor 68070 and 32213), imaged bands on a Licor Odyssey CLx Imager, and quantified intensity using ImageJ.

### Chromatin-IP and Sequencing

We performed ChIP-seq experiments using a standard protocol adapted from Alekseyenko et al. (2006). Briefly, ∼2 ml of adult flash-frozen flies were dissected on dry ice, and heads and thoraces were used to fix and isolate chromatin. Following chromatin isolation, we spiked in 60 μl of chromatin prepared from female *D. miranda* larvae (∼1 μg of chromatin); for replicate experiments, we used new preparations of *D. melanogaster* chromatin and the same *D. miranda* chromatin spike. We then performed immunoprecipitation using 4 μl of one of the following antibodies: H3K9me2 (Abcam ab1220), H3K9me3 (Abcam ab8898), and H3K4me3 (Abcam ab8580).

After reversing the crosslinks and isolating DNA, we constructed sequencing libraries using the BIOO NextFlex sequencing kit. Sequencing was performed at the Vincent J. Coates Genomic Sequencing Laboratory at UC Berkeley, supported by NIH S10 Instrumentation Grants S10RR029668 and S10RR027303. We performed 50-bp single-read sequencing for our input and H3K4me3 libraries, and 100-bp paired-end sequencing for the H3K9me2 and H3K9me3 libraries, due to their higher repeat content.

For H3K4me3, Pearson correlation values between the five karyotypes is very high, and the magnitude of difference between the samples is low (supplementary table S6, Supplementary Material online). For the two heterochromatin marks, Pearson correlation values between the two marks were generally high for all samples, and overlap of the top 40% of 5-kb windows was similarly high for all samples (supplementary table S6, Supplementary Material online). Additionally, we obtained replicates for H3K9me3 for all samples except XX female, which has extremely high correlation values between H3K9me2 and H3K9me3. The unspiked replicate data for H3K9me3 correlate well with the *D. miranda* chromatin spike data that were used for the bulk of our analyses (supplementary table S6, Supplementary Material online).

We also generated replicate ChIP-seq data for H3K9me3 from X0, XXY, and XYY individuals using a different attached X stock, 4248, and a different ChIP-seq protocol, ULI-NChIP-seq, based on Brind'Amour et al. (2015). Briefly, four flies from each of the three karyotypes were collected, heads were dissected, and along with a single *D. miranda* head, were homogenized in PBS and spun at 500 g to isolate nuclei. MNase digestion was performed at 37 °C for 5 min, at which point the reaction was stopped by the addition of 10% 100 μM ethylenediaminetetraacetic acid and incubated for 1 h in complete immunoprecipitation buffer (20 mM Tris–HCl pH 8.0, 2 mM ethylenediaminetetraacetic acid, 150 mM NaCl, 0.1% Triton X-100, 1 mM Phenylmethylsulfonyl Fluoride, and 1× protease inhibitors). Samples were then incubated overnight at 4 °C with 1 μg of H3K9me3 antibody and 10 μl of Dynabeads (Life Technologies 1006D). Libraries were then prepared using the BIOO NextFlex sequencing kit and sequenced at the Vincent J. Coates Genomic Sequencing Laboratory at UC Berkeley. An overview of all ChIP-seq data generated can be found in supplementary table S3, Supplementary Material online.

### RNA Extraction and RNA-seq

We collected mated males and females of the various karyotypes, aged them for 8 days, and dissected and pooled five heads from each karyotype. A replicate set of individuals was collected from independent vials for the wildtype Canton-S, and independent crosses for the X0, XXY, and XYY individuals. We then extracted RNA and prepared stranded total RNA-seq libraries using Illumina’s TruSeq Stranded Total RNA Library Prep kit with Ribo-Zero ribosomal RNA reduction chemistry, which depletes the highly abundant ribosomal RNA transcripts (Illumina RS-122-2201). We performed 50-bp single-read sequencing for all total RNA libraries at the Vincent J. Coates Genomic Sequencing Laboratory at UC Berkeley. An overview of all RNA-seq data generated can be found in supplementary table S7, Supplementary Material online.

### Mapping of Sequencing Reads and Data Normalization

For all *D. melanogaster* alignments, we used Release 6 of the genome assembly and annotation (Hoskins et al. 2015). For all ChIP-seq data sets, we used Bowtie2 (Langmead and Salzberg 2012) to map reads to the genome, using the parameters “-D 15 –R 2 –N 0 –L 22 –i S , 1,0.50 –no-1mm-upfront,” which allowed us to reduce cross-mapping to the *D. miranda* genome to ∼2.5% of 50-bp reads, and 1% of 100-bp paired-end reads. We also mapped all ChIP-seq data sets to the *D. miranda* genome assembly (Ellison and Bachtrog 2013) to calculate the proportion of each library that originated from the spiked-in *D. miranda* chromatin versus the *D. melanogaster* sample. To correct for variable coverage based on GC content for our heterochromatin ChIPs (GC content bias), we used a shell script written by Flynn et al. (2017) to calculate correction factors following Benjamini and Speed (2012). In particular, we calculated the average coverage of uniquely mappable regions of the genome, binned by GC content in 5% intervals. These values correspond to the expected coverage across the genome based on GC content. To normalize coverage of repetitive elements based on GC content, we divided the observed coverage of the repeat by the expected coverage based on the GC content of the repetitive element. To normalize coverage of the genome by GC content, we divided the observed coverage by the expected coverage based on the GC content of the 5-kb region.

To calculate ChIP signal, we first calculated the coverage across 5-kb windows for both the ChIP and the input and then normalized by the total library size, including reads that map to both *D. melanogaster* and the *D. miranda* spike. We then calculated the ratio of ChIP coverage to input coverage and normalized by the ratio of *D. melanogaster* reads to *D. miranda* reads in the ChIP library, and then by the ratio of *D. melanogaster* reads to *D. miranda* reads in the input, to account for differences in the ratio of sample to spike present before immunoprecipitation. For replicate ChIPs without a spike-in (supplementary fig. S8, Supplementary Material online), we simply normalized the coverage in the IP library by the input coverage. Note that this normalization procedure accounts for differences in ploidy as well as genome size by using a ratio of ChIP coverage to input coverage (see supplementary fig. S7, Supplementary Material online).

### Gene Expression Analysis

We first mapped RNA-seq reads to the rDNA scaffold in the Release 6 version of the genome and removed all reads that mapped to this scaffold, as differences in rRNA expression are likely to be technical artifacts from the total RNA library preparation. We then mapped the remaining reads to the Release 6 version of the *D. melanogaster* genome using STAR (Dobin et al. 2013) with default parameters. We then counted reads mapping to each transcript using the FeatureCounts of Subread (Liao et al 2014). Gene counts were then imported into DESeq2 for differential expression analysis (Love et al 2014), using the two replicates for each karyotype to calculate log fold change and *P* value estimates. GO analysis was performed using GOrilla using ranked lists of differentially expressed genes (Eden et al. 2009). A list of expression values for all genes is provided in supplementary appendix S1, Supplementary Material online.

### Repeat Libraries

We used two approaches to quantify expression of repeats. Our first approach was based on consensus sequences of known repetitive elements that were included in the Release 6 version of the *D. melanogaster* genome and are available on FlyBase. These included consensus sequences for 125 TEs. We also added the consensus sequences of three known satellite sequences (Dodeca, Responder, and 359), to include larger non-TE repetitive sequences in our repeat analyses.

We were particularly interested in misregulation of the Y chromosome, which is poorly assembled. We therefore assembled repetitive elements de novo from male and female genomic DNA reads using RepARK (Koch et al. 2014), setting a manual threshold for abundant kmers of five times the average genome coverage, which corresponds to a repetitive sequence occurring at least five times in the genome. To identify male-specific repeats, we mapped male and female genomic reads back to our de novo assembled repeats and identified repeats that had high coverage in males and either no coverage or significantly lower coverage in females (supplementary fig. S27, Supplementary Material online). After filtering in this way, we obtained 101 male-specific repeats comprising 13.7 kb of sequence, with a median repeat size of 101 bp. This male-specific repeat library is provided in supplementary appendix S2, Supplementary Material online.

## Acknowledgements

This work was funded by National Institutes of Health grants (nos. R01GM076007, R01GM101255 and R01AG057029) to DB.

## 

All the data generated have been deposited at GenBank under BioProject PRJNA594555.

## Supplementary Material

msaa082_supplementary_data
