# Supplementary material for: The Drosophila Y Chromosome Affects Heterochromatin Integrity Genome-Wide
Source: Mol Biol Evol. 2020 Mar 25;37(10):2808–24. doi: 10.1093/molbev/msaa082 (PMC7530609; doi:10.1093/molbev/msaa082)
Supplement: msaa082_supplementary_data [file msaa082_supplementary_data.zip › Supplements_reorganized.pdf]

Supplementary Information

A. Fly stocks and crosses

We used the wildtype Canton-S stock, and the *D. melanogaster* stock number 2549 from the Bloomington Stock Center, which has a compound reversed metacentric X chromosome (C(1)RM) or a hetero-compound chromosome with the X chromosome inserted between the two arms of the Y chromosome (C(1;Y)), to generate X0, XXY and XYY (see **Figure S1** for crossing scheme). We also generated XO, XXY, and XYY individuals using a different attached X/Y stock (4248), to repeat some of our ChIP experiments in a different background (**Figure S11**).

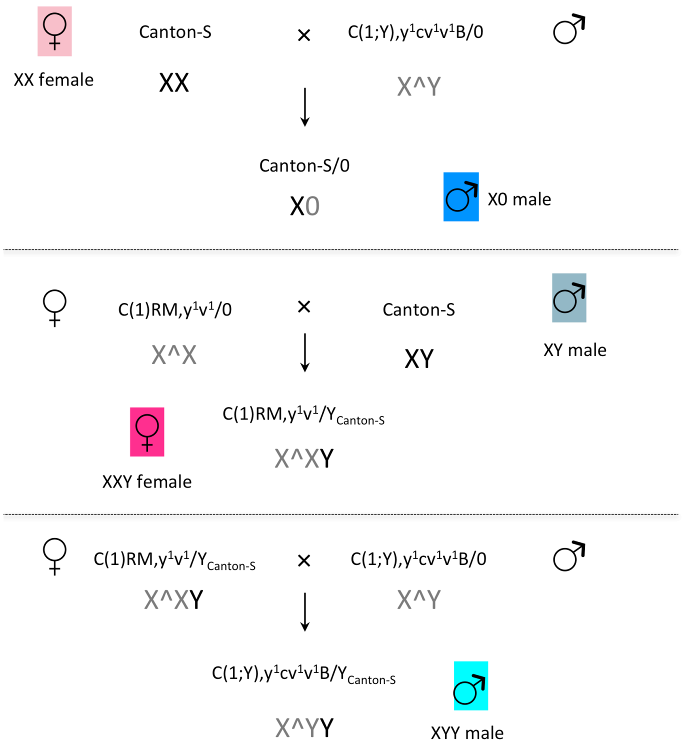

Figure S1. Crossing scheme to generate X0, XXY and XYY flies.

B. Genome size estimates

We used flow cytometry (**Table S1**) and a sequencing-based method (**Figure S2**) to estimate the repeat content of the different strains investigated.

Table S1. Flow cytometry estimates of genome sizes for flies with different karyotypes. Shown are the measurements from 3 independent replicates.

	Replicate 1	Replicate 2	Replicate 3	Mean	SE
CantonS XX	177.6	177.2	180.4	178.4	1.8
CantonS XY	179.9	178	178.5	178.8	1
XO (2549 x CantonS)	159.7	157.9	161.3	159.6	1.7
XXY (2549 x CantonS)	195.8	192.8	195.7	194.8	1.7
XYY (2549 x CantonS)	194.5	200.5	197.4	197.4	3

We also performed an independent characterization of repetitive elements using *de novo* assembly of repeats with dnaPipeTE (Goubert *et al.* 2015). We extracted genomic DNA from single whole flies (**Table S2**) and estimated the amount of unique DNA in each strain. Note that sequencing-based approaches greatly underestimate the amount of repetitive DNA (due to biases in library construction; see Wei *et al.* 2014), but relative repeat abundance is qualitatively similar between the flow cytometry and the dnaPipeTE analysis (**Figure S2**).

Table S2. Overview of genomic data analyzed

Karyotype	Genotype	Data type	Tissue	Figure	no. reads	SRA Accession
XX	Canton-S wt	genomic DNA	whole fly	Fig. S1, S18	24225757	TBA
XY	Canton-S wt	genomic DNA	whole fly	Fig. S1, S18	26008180	TBA
X0	2549 strain	genomic DNA	whole fly	Fig. S1, S19	18275359	TBA
XXY	2549 strain	genomic DNA	whole fly	Fig. S1, S19	19548531	TBA
XYY	2549 strain	genomic DNA	whole fly	Fig. S1, S19	19672692	TBA

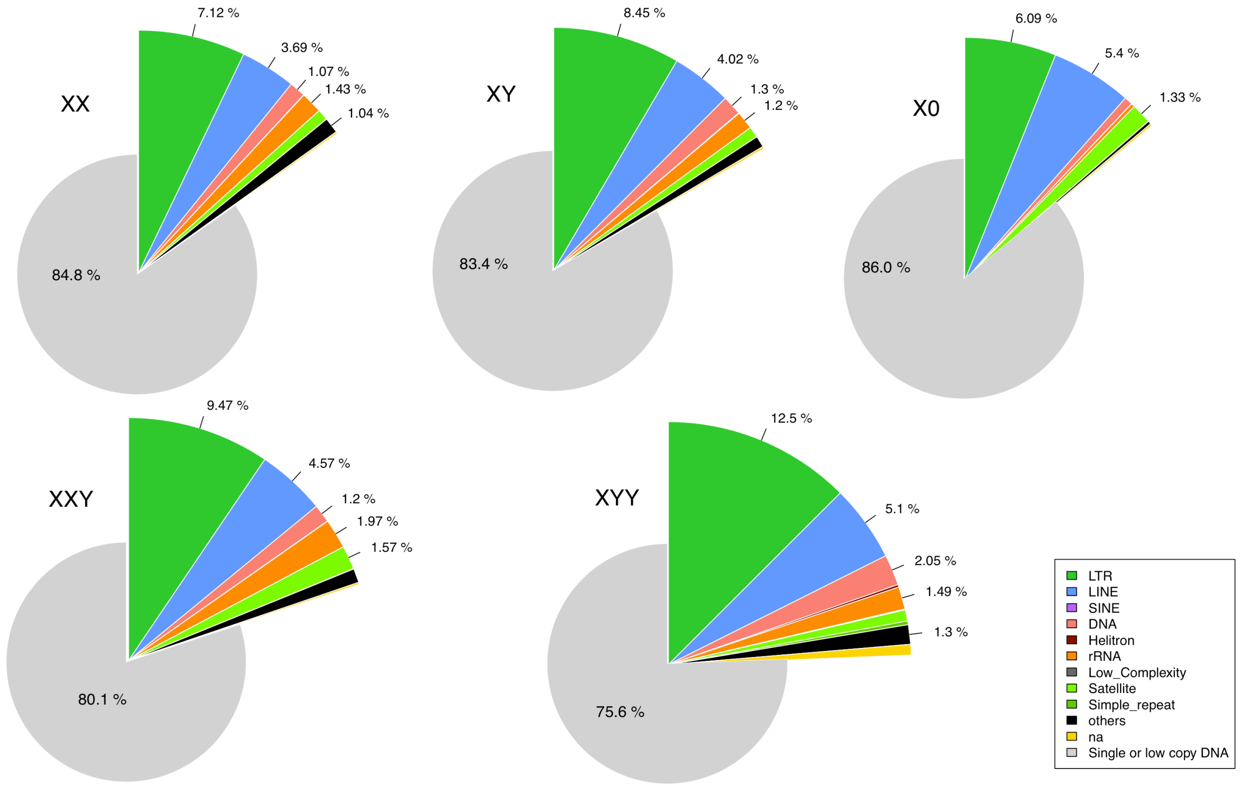

Figure S2. De-novo estimates (dnaPipeTE) of repetitive DNA among different karyotypes.

C. Chromatin profiling

We generated a variety of datasets to test the hypothesis that the Y chromosome acts to modulate heterochromatin integrity and gene expression genome-wide. **Table S3** gives an overview of all the ChIP data analyzed, and details where they are displayed throughout the manuscript and supplementary materials. **Table S4** gives an overview of mapping statistics for the different libraries.

Table S3. Overview of ChIP data analyzed

Karyotype	Genotype	Data type	Antibody	Spike-in	Tissue	Figure	SRA Accession
XX	Canton-S wt	ChIP-seq	H3K4me3	*D. miranda*	head/thorax	Fig. 2	TBA
XY	Canton-S wt	ChIP-seq	H3K4me3	*D. miranda*	head/thorax	Fig. 2	TBA
X0	2549 strain	ChIP-seq	H3K4me3	*D. miranda*	head/thorax	Fig. 2	TBA
XXY	2549 strain	ChIP-seq	H3K4me3	*D. miranda*	head/thorax	Fig. 2	TBA
XYY	2549 strain	ChIP-seq	H3K4me3	*D. miranda*	head/thorax	Fig. 2	TBA
XX	Canton-S wt	ChIP-seq	H3K9me2	*D. miranda*	head/thorax	Fig. 3	TBA
XY	Canton-S wt	ChIP-seq	H3K9me2	*D. miranda*	head/thorax	Fig. 3	TBA
X0	2549 x Canton-S	ChIP-seq	H3K9me2	*D. miranda*	head/thorax	Fig. 3	TBA
XXY	2549 x Canton-S	ChIP-seq	H3K9me2	*D. miranda*	head/thorax	Fig. 3	TBA
XYY	2549 x Canton-S	ChIP-seq	H3K9me2	*D. miranda*	head/thorax	Fig. 3	TBA
XX	Canton-S wt	ChIP-seq	H3K9me3	*D. miranda*	head/thorax	Fig. 4	TBA
XY	Canton-S wt	ChIP-seq	H3K9me3	*D. miranda*	head/thorax	Fig. 4	TBA
X0	2549 x Canton-S	ChIP-seq	H3K9me3	*D. miranda*	head/thorax	Fig. 4	TBA
XXY	2549 x Canton-S	ChIP-seq	H3K9me3	*D. miranda*	head/thorax	Fig. 4	TBA
XYY	2549 x Canton-S	ChIP-seq	H3K9me3	*D. miranda*	head/thorax	Fig. 4	TBA
XX	Canton-S wt	ChIP-seq	Input	*D. miranda*	head/thorax	Fig. 2-4	TBA
XY	Canton-S wt	ChIP-seq	Input	*D. miranda*	head/thorax	Fig. 2-4	TBA
X0	2549 x Canton-S	ChIP-seq	Input	*D. miranda*	head/thorax	Fig. 2-4	TBA
XXY	2549 x Canton-S	ChIP-seq	Input	*D. miranda*	head/thorax	Fig. 2-4	TBA
XYY	2549 x Canton-S	ChIP-seq	Input	*D. miranda*	head/thorax	Fig. 2-4	TBA
XX	Canton-S wt	ChIP-seq	H3K4me3	none	head/thorax	Fig. S6	TBA
XY	Canton-S wt	ChIP-seq	H3K4me3	none	head/thorax	Fig. S6	TBA
X0	2549 strain	ChIP-seq	H3K4me3	none	head/thorax	Fig. S6	TBA
XXY	2549 strain	ChIP-seq	H3K4me3	none	head/thorax	Fig. S6	TBA
XYY	2549 strain	ChIP-seq	H3K4me3	none	head/thorax	Fig. S6	TBA
XX	Canton-S wt	ChIP-seq	H3K9me3	none	head/thorax	Fig. S7	TBA
XY	Canton-S wt	ChIP-seq	H3K9me3	none	head/thorax	Fig. S7	TBA
X0	2549 x Canton-S	ChIP-seq	H3K9me3	none	head/thorax	Fig. S7	TBA
XXY	2549 x Canton-S	ChIP-seq	H3K9me3	none	head/thorax	Fig. S7	TBA
XYY	2549 x Canton-S	ChIP-seq	H3K9me3	none	head/thorax	Fig. S7	TBA
XX	Canton-S wt	ChIP-seq	Input	none	head/thorax	Fig S6,S7	TBA
XY	Canton-S wt	ChIP-seq	Input	none	head/thorax	Fig S6,S7	TBA
X0	2549 strain	ChIP-seq	Input	none	head/thorax	Fig S6,S7	TBA
XXY	2549 strain	ChIP-seq	Input	none	head/thorax	Fig S6,S7	TBA
XYY	2549 strain	ChIP-seq	Input	none	head/thorax	Fig S6,S7	TBA
X0	4248 x Canton-S	Uli-ChIP-seq	H3K9me3	*D. miranda*	head/thorax	Fig. S8	TBA
XXY	4248 x Canton-S	Uli-ChIP-seq	H3K9me3	*D. miranda*	head/thorax	Fig. S8	TBA
XYY	4248 x Canton-S	Uli-ChIP-seq	H3K9me3	*D. miranda*	head/thorax	Fig. S8	TBA
X0	4248 x Canton-S	Uli-ChIP-seq	Input	*D. miranda*	head/thorax	Fig. S8	TBA
XXY	4248 x Canton-S	Uli-ChIP-seq	Input	*D. miranda*	head/thorax	Fig. S8	TBA
XYY	4248 x Canton-S	Uli-ChIP-seq	Input	*D. miranda*	head/thorax	Fig. S8	TBA

Table S4 Mapping statistics of ChIP data

Sample	Total reads	Reads map to *D. mel*	Reads map to *D. mir*	Total INPUT reads	Input reads map *D. mel*	Input reads map *D. mir*
**H3K4me3 ChIP**						
CantonS F	46,914,779	32,273,669	14,292,401	37,413,699	25,010,775	12,023,243
CantonS M	17,194,149	9,718,592	7,464,505	25,053,492	14,486,914	10,387,226
2549 XO	34,946,716	22,149,946	12,687,551	31,339,408	18,714,779	12,162,612
2549 XXY	18,609,198	10,139,961	7,341,215	20,556,162	9,699,559	9,643,507
2549 XYY	30,996,481	18,977,564	11,716,942	30,628,442	16,728,779	13,660,078
**H3K9me2 ChIP**						
CantonS F	25,655,496	15,063,349	9,851,094	22,323,023	12,325,622	8,876,560
CantonS M	25,507,652	12,691,329	12,004,466	25,053,492	14,486,914	10,387,226
2549 XO	28,811,020	7,573,255	17,427,157	19,053,148	5,169,705	12,972,035
2549 XXY	34,668,602	15,497,299	16,275,475	20,556,162	9,699,559	9,643,507
2549 XYY	34,833,102	17,616,945	15,572,868	30,628,442	16,728,779	13,660,078
**H3K9me3 ChIP**						
CantonS F	47,525,730	29,050,892	14,639,179	22,323,023	12,325,622	8,876,560
CantonS M	26,092,010	17,809,890	7,721,998	25,053,492	14,486,914	10,387,226
2549 XO	30,942,218	12,276,089	15,858,448	19,053,148	5,169,705	12,972,035
2549 XXY	27,755,576	17,216,581	8,542,009	20,556,162	9,699,559	9,643,507
2549 XYY	32,191,604	21,022,577	9,709,157	30,628,442	16,728,779	13,660,078
**H3K4me3 - no spike**						
CantonS F	11,789,851	7,202,373	N/A	10,322,202	7,250,933	N/A
CantonS M	16,626,442	7,829,518	N/A	13,260,661	7,294,178	N/A
2549 XO	26,303,999	15,479,471	N/A	15,344,578	13,421,899	N/A
2549 XXY	25,954,152	20,444,321	N/A	20,483,190	10,471,961	N/A
2549 XYY	21,367,074	19,926,377	N/A	23,521,804	21,761,800	N/A
**H3K9me3 - no spike**						
CantonS F	47,525,730	29,050,892	N/A	10,322,202	7,250,933	N/A
CantonS M	26,092,010	17,809,890	N/A	13,260,661	7,294,178	N/A
2549 XO	45,401,828	14,937,945	N/A	15,344,578	13,421,899	N/A
2549 XXY	44,728,192	15,878,414	N/A	20,483,190	10,471,961	N/A
2549 XYY	17,239,488	15,998,407	N/A	23,521,804	21,761,800	N/A
**H3K9me3 ULI-ChIP**						
4248 XO	61,279,122	24,019,850	34,509,125	48,602,150	23,558,961	23,225,464
2549 XXY	60,587,174	49,491,822	10,311,462	63,182,508	54,068,049	8,066,212
2549 XYY	66,451,452	46,813,218	17,940,085	73,249,354	55,131,186	16,523,221

C1. Robustness of ChIP analysis

We show that our inferences are robust to window size (**Figure S3-S6**), that normalization accounts for differences in ploidy of sex chromosomes (**Figure S7**), and that our inferences are robust with regards to different normalization strategies or multiple mapping of reads (**Figure S8**). Signal for our histone marks are typically highly correlated across samples or replicates (**Table S5, S6).**

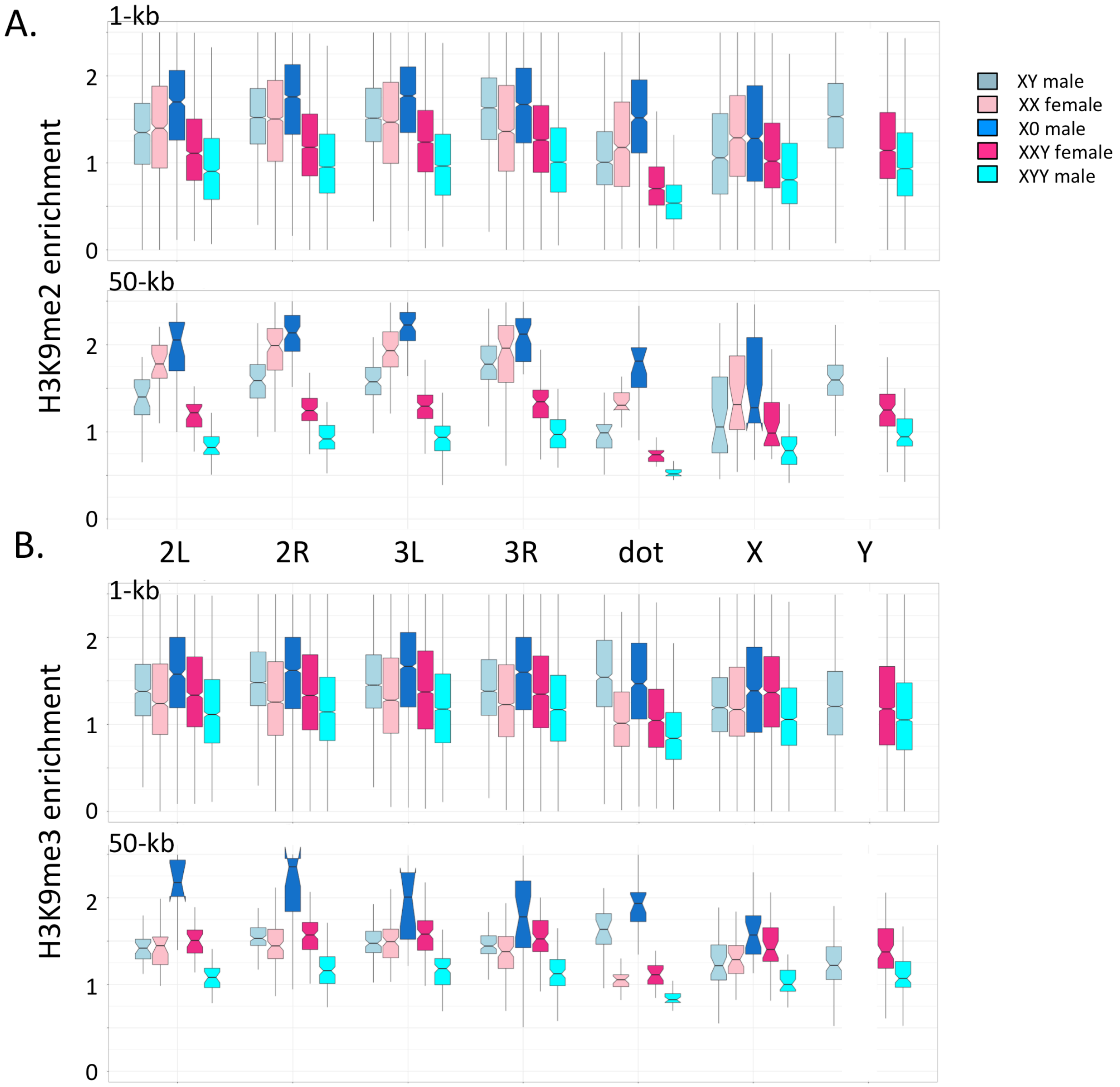

Figure S3. . Enrichment of **A.** H3K9me2, **B.** H3K9me3 using 1-kb and 50-kb windows (top & bottom). The box plots show the ChIP signal for all windows in different chromosomal regions, with boxes extending from the first to the third quartile and whiskers to the most extreme data point within 1.5 times the interquantile range.

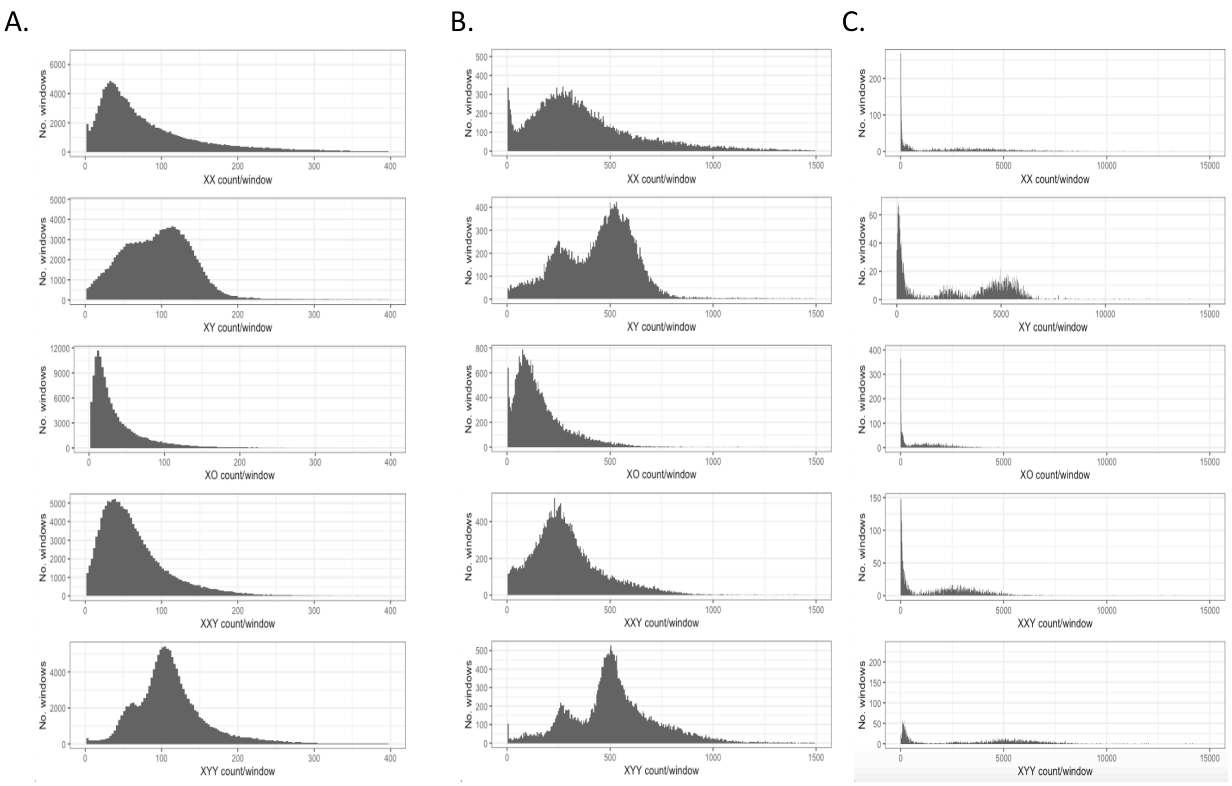

Figure S4. Uniquely mapping Input reads across the genome for the different karyotypes investigated using **A.** 1-kb, **B**. 5-kb, **C.** 50-kb windows.

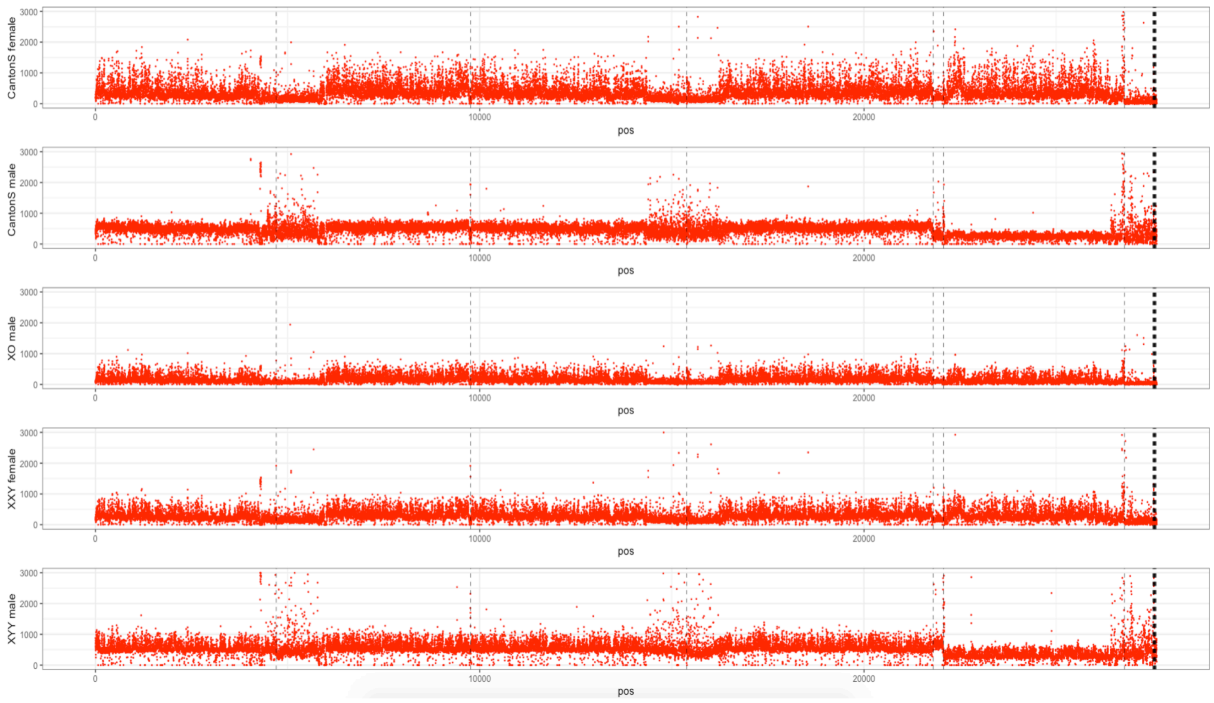

Figure S5. Uniquely mapping Input reads across the genome for the different karyotypes investigated (5-kb windows).

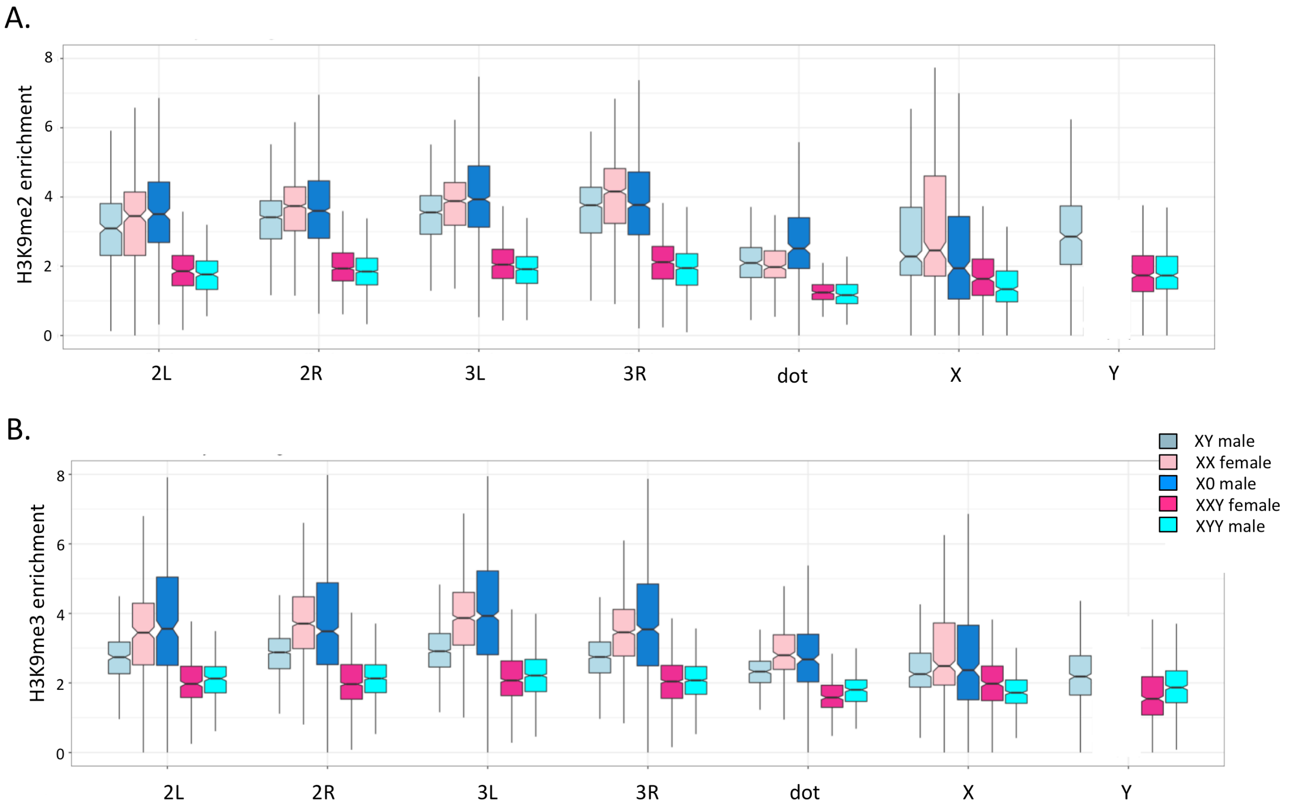

Figure S6. Enrichment of **A.** H3K9me2, **B.** H3K9me3 using 5-kb windows, removing windows with <10 Input reads mapping. The box plots show the ChIP signal for all 5-kb windows in different chromosomal regions, with boxes extending from the first to the third quartile and whiskers to the most extreme data point within 1.5 times the interquantile range.

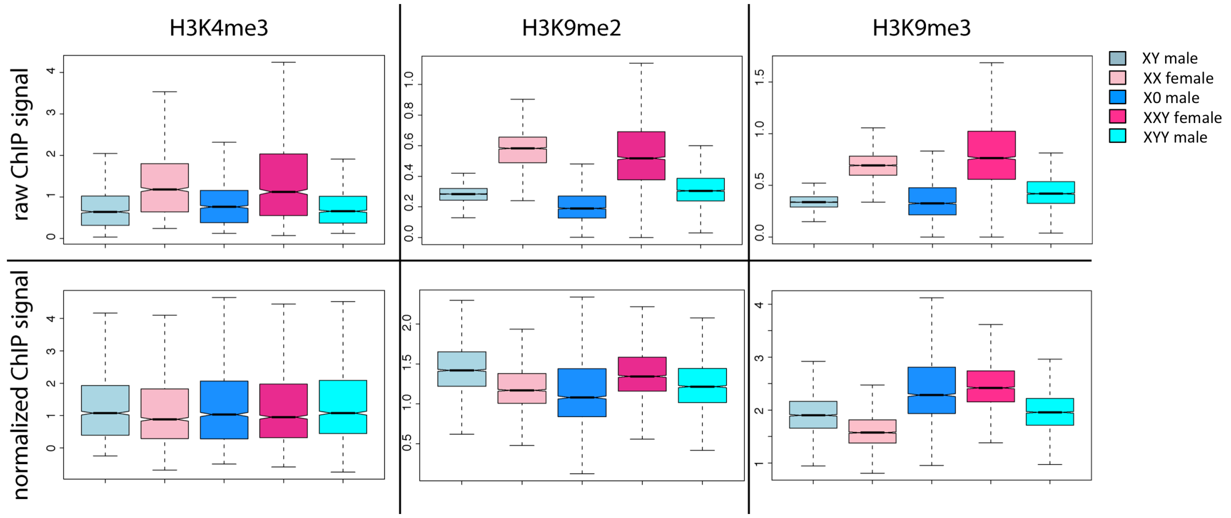

Figure S7. Normalization accounts for differences in ploidy of sex chromosomes. In the upper boxplots, we show the raw ChIP signal in genes on the X chromosome, where we know that there is a two-fold difference in ploidy between male and female karyotypes. In the lower boxplot, we plot the normalized signal in genes on the X chromosome, demonstrating that our normalization method corrects for differences in signal driven by differences in ploidy for all three histone modifications assayed.

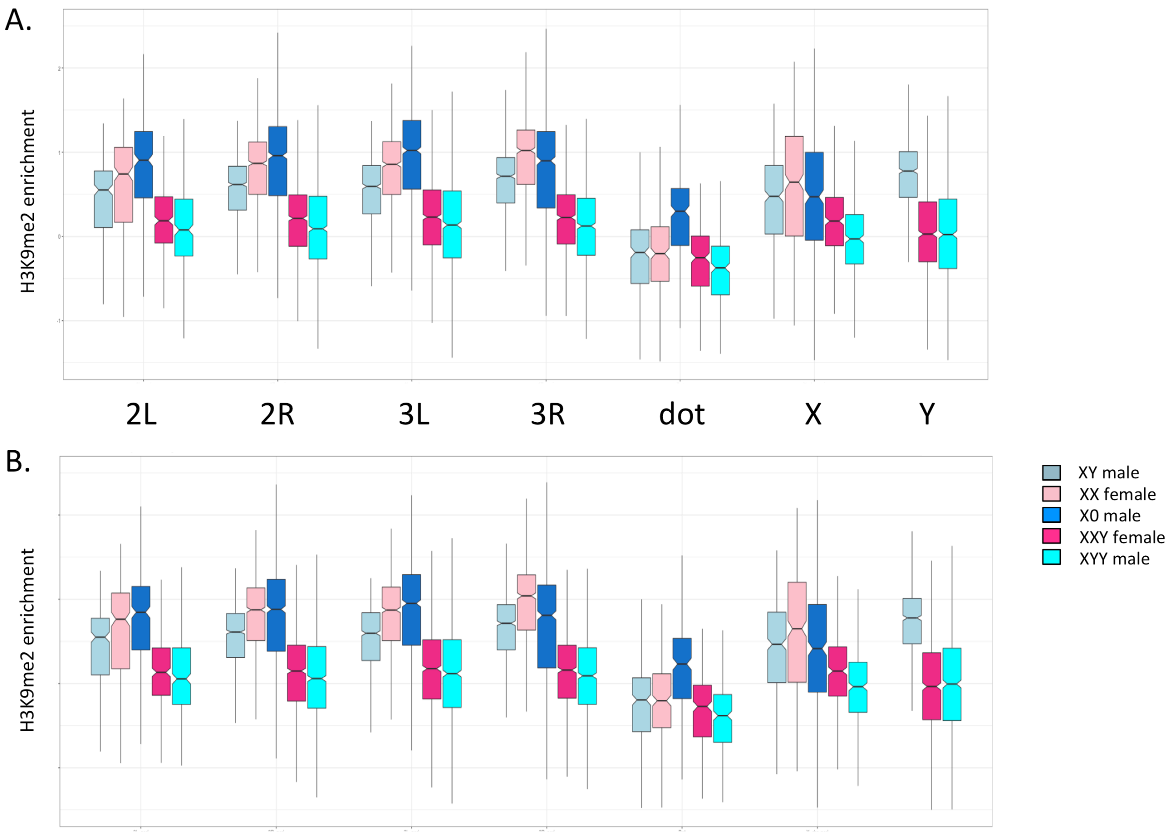

Figure S8. H3K9me2 enrichment using **A.** a normalization strategy based on a linear regression model **B.** only uniquely mapping reads at heterochromatic regions (pericentromere of chr2, chr3, X, the dot and Y chromosome). The box plots show the ChIP signal for all 5kb windows in different chromosomal regions, with boxes extending from the first to the third quartile and whiskers to the most extreme data point within 1.5 times the interquantile range.

Table S5. Pearson correlation coefficients of signal of *D. miranda* spike for the H3K4me3, H3K9me2 and K3K9me3 ChIP's. The signal is calculated as the ratio of reads from the immunoprecipitation (normalized by library size) to reads from the input (normalized by library size) and multiplied by the normalization factor, as described in the methods. Correlation coefficients are calculated based on normalized ChIP-seq signal in 5-kb non-overlapping windows. Values below the diagonal are before GC correction, and below the diagonal are after GC correction.

	H3K4me3					H3K9me2					H3K9me3				
	XY	XX	X0	XXY	XYY	XY	XX	X0	XXY	XYY	XY	XX	X0	XXY	XYY
XY		0.87	0.97	0.91	0.92		0.95	0.77	0.75	0.84		0.78	0.68	0.64	0.80
XX	0.87		0.89	0.90	0.90	0.99		0.82	0.73	0.78	0.94		0.75	0.41	0.62
X0	0.82	0.96		0.91	0.94	0.72	0.72		0.62	0.6	0.79	0.81		0.58	0.63
XXY	0.82	0.71	0.67		0.92	0.81	0.8	0.95		0.76	0.8	0.76	0.95		0.71
XYY	0.94	0.85	0.84	0.78		0.95	0.94	0.86	0.92		0.94	0.92	0.90	0.90	

Table S6. Pearson correlation coefficients for different ChIP experiments. Shown are correlation coefficients of H3K9me3 signal across different karyotypes. Correlation coefficients are calculated based on normalized ChIP-seq signal in 5-kb non-overlapping windows.

	XY	XX	X0	XXY	XYY
Correlation of H3K4me3 across samples					
XX	0.90				
X0	0.93	0.96			
XXY	0.87	0.82	0.83		
XYY	0.89	0.88	0.89	0.75	
H3K9me2 signal vs. H3K9me3 signal					
Pearson correlcation	0.74	0.90	0.67	0.53	0.76
overlap of top 40% windows	0.69	0.72	0.60	0.66	0.69
Unspiked vs. spiked H3K9me3 replicates					
	0.76	n/a	0.68	0.57	0.90

C2. Biological replicates

We collected independent biological replicate ChIP data without a *D. miranda* chromatin spike for the H3K4me3 and H3K9me3 histone mark (**Figure S9, S10**), and we also generated replicate ChIP-seq data for H3K9me3 from XO, XXY, and XYY individuals using a different attached X stock, 4248 (**Figure S11**).

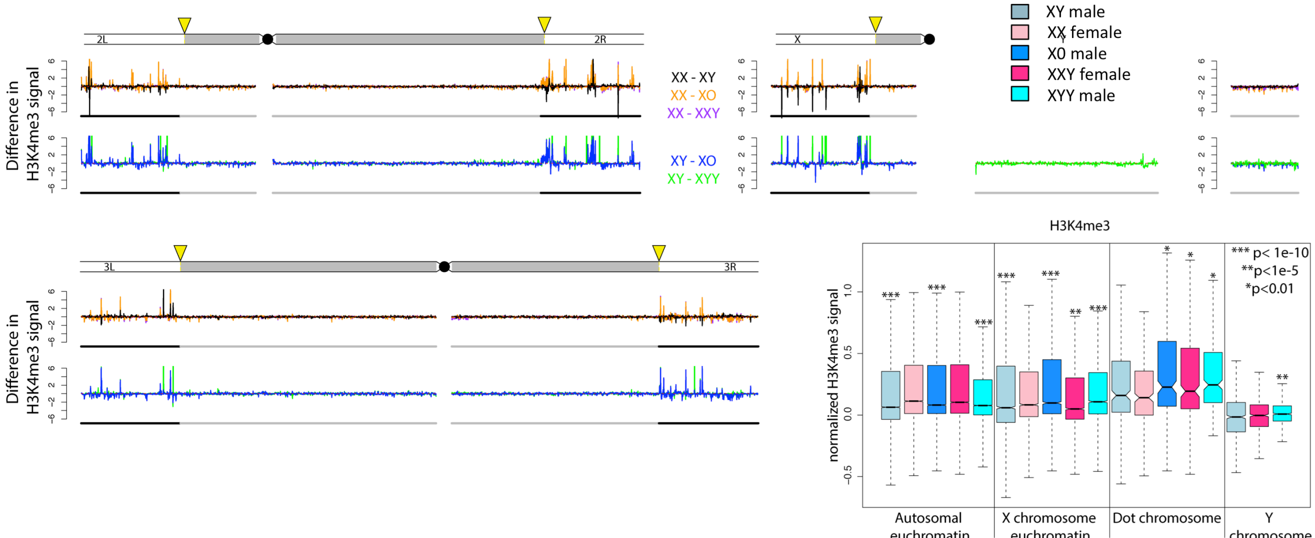

Figure S9. Biological replicate for H3K4me3 (sample without a spike).

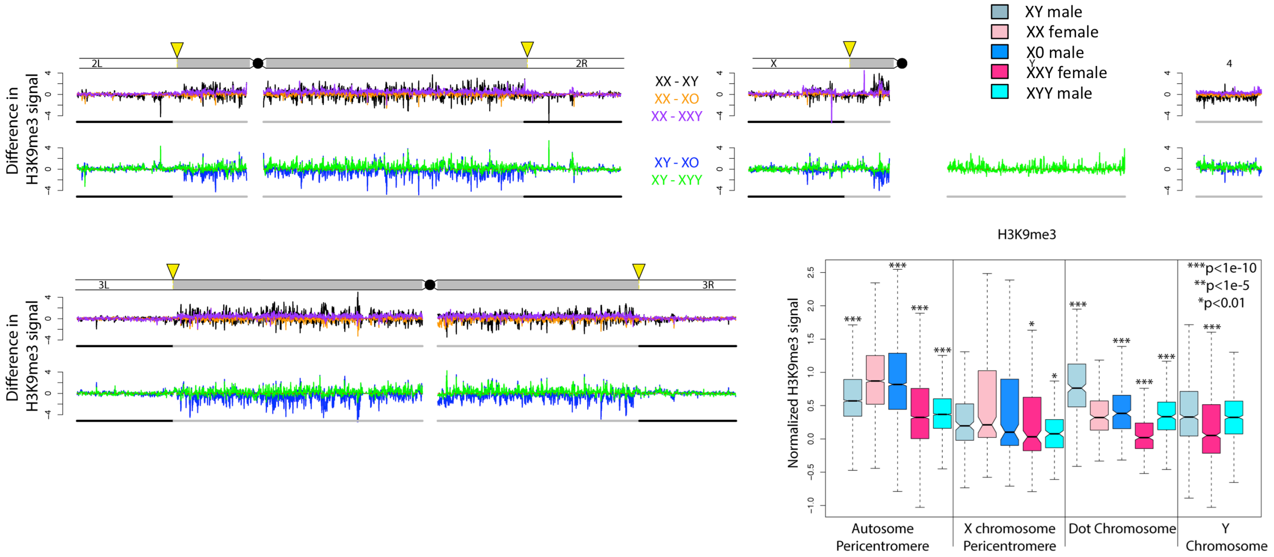

Figure S10. Biological replicate for H3K9me3 (sample without a spike).

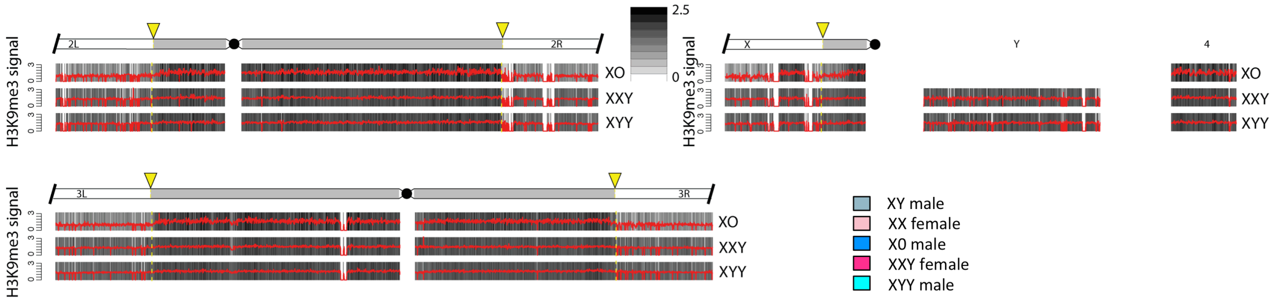

Figure S11. H3K9me3 ChIPs for replicate karyotypes with aberrant sex chromosomes. X0/XXY and XYY flies were generated by crossing Canton-S flies with the 4248 stock (a stock with a different attached-X, attached X-Y chromosome). For legend see Fig. 2-4.

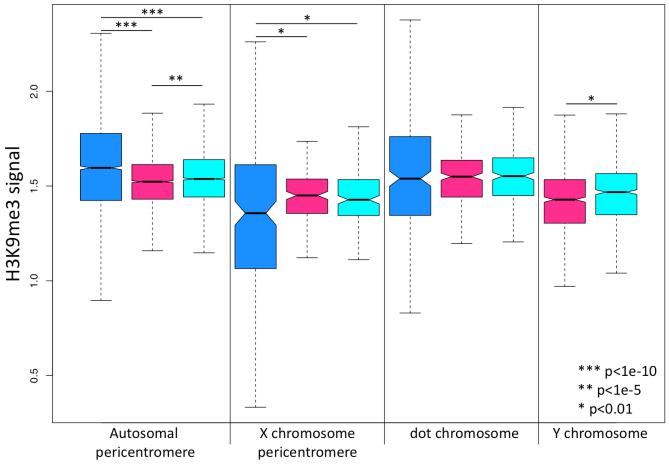

C3. Overall levels of heterochromatin marks

Western plots suggest that absolute levels of heterochromatin marks are similar among karyotypes (**Figure S12**).

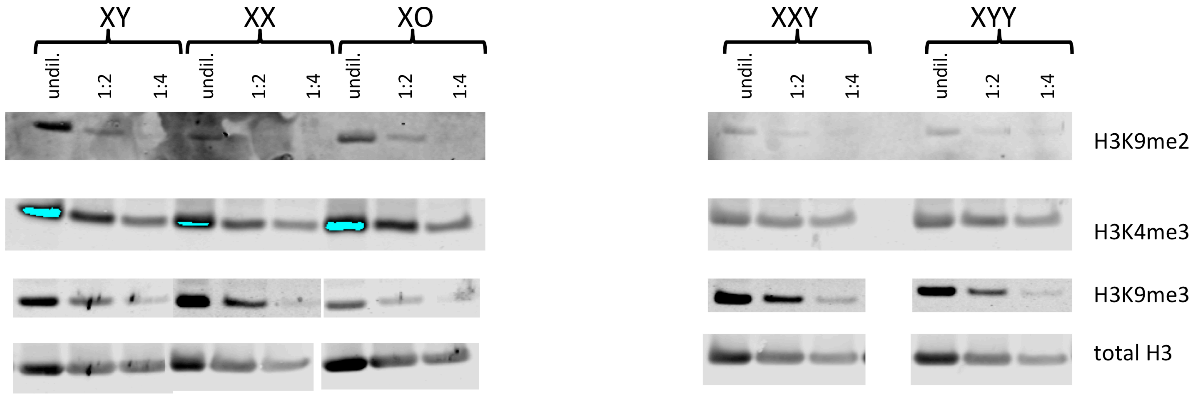

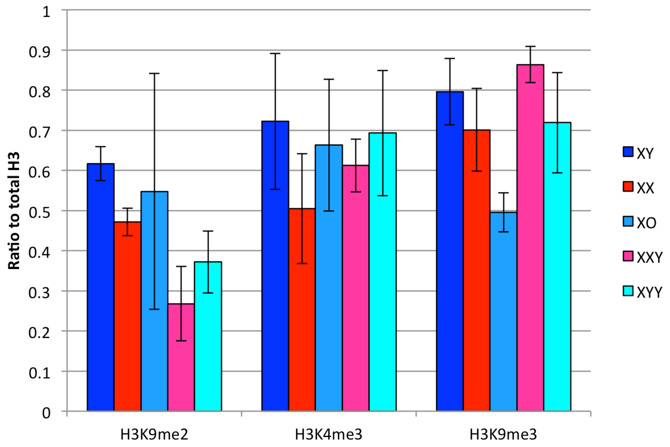

Figure S12. Western blots for H3K9me2, H3K9me3 and H3K4me3 for the different karyotypes. We normalized the Western signal for all histone modifications by signal for total H3, and averaged across dilutions that were within the linear signal range from two replicated experiments (representative blots are shown).

C4. Genome-wide enrichment plots

Genome-wide enrichment plots for the three histone marks are shown in **Figure S13-15**. The plots were made using the same data and normalization procedures described in Figures 2-4.

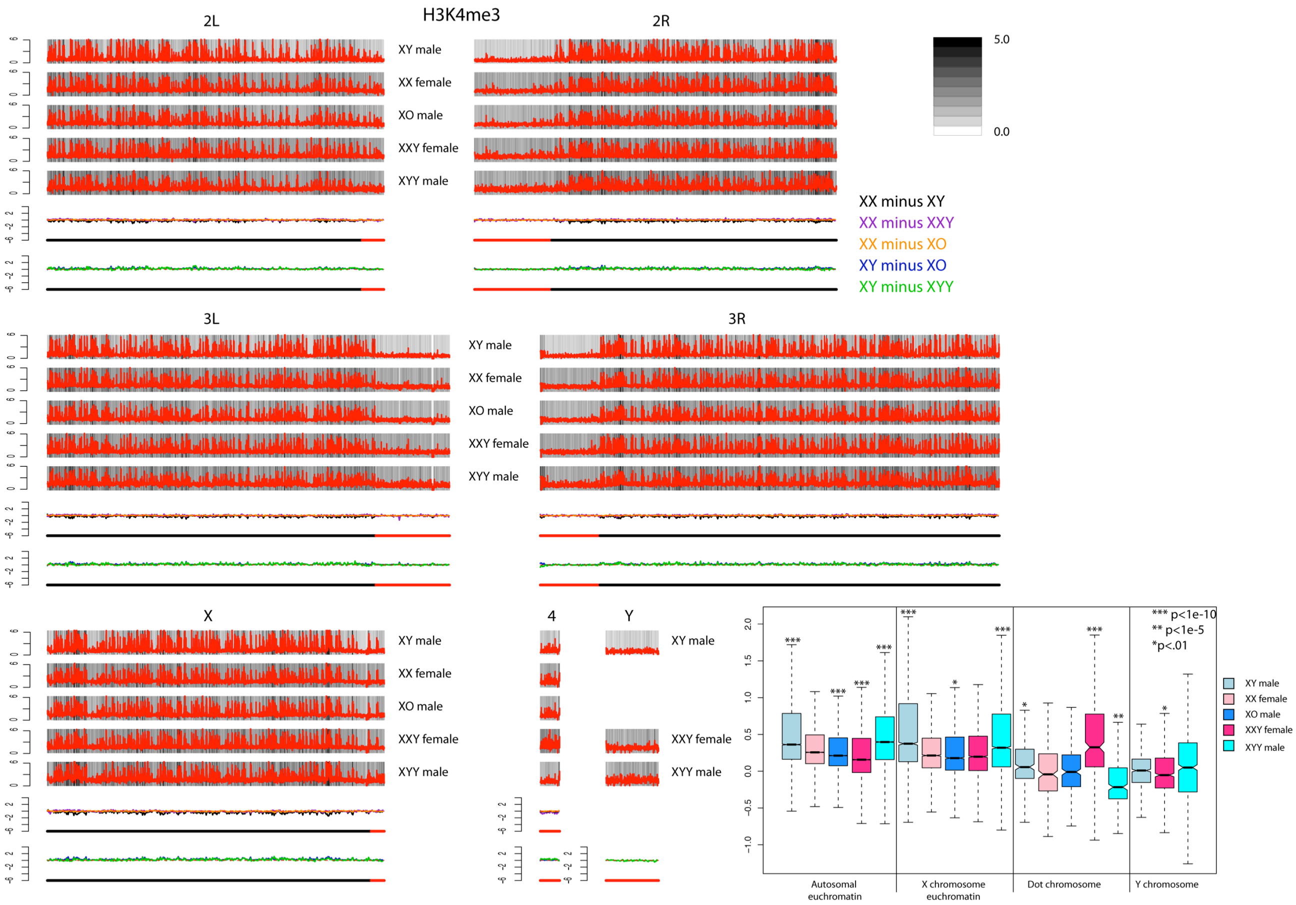

Figure S13. Genome-wide enrichment of H3K4me3 for *D. melanogaster* strains with different karyotype along the different chromosome arms.

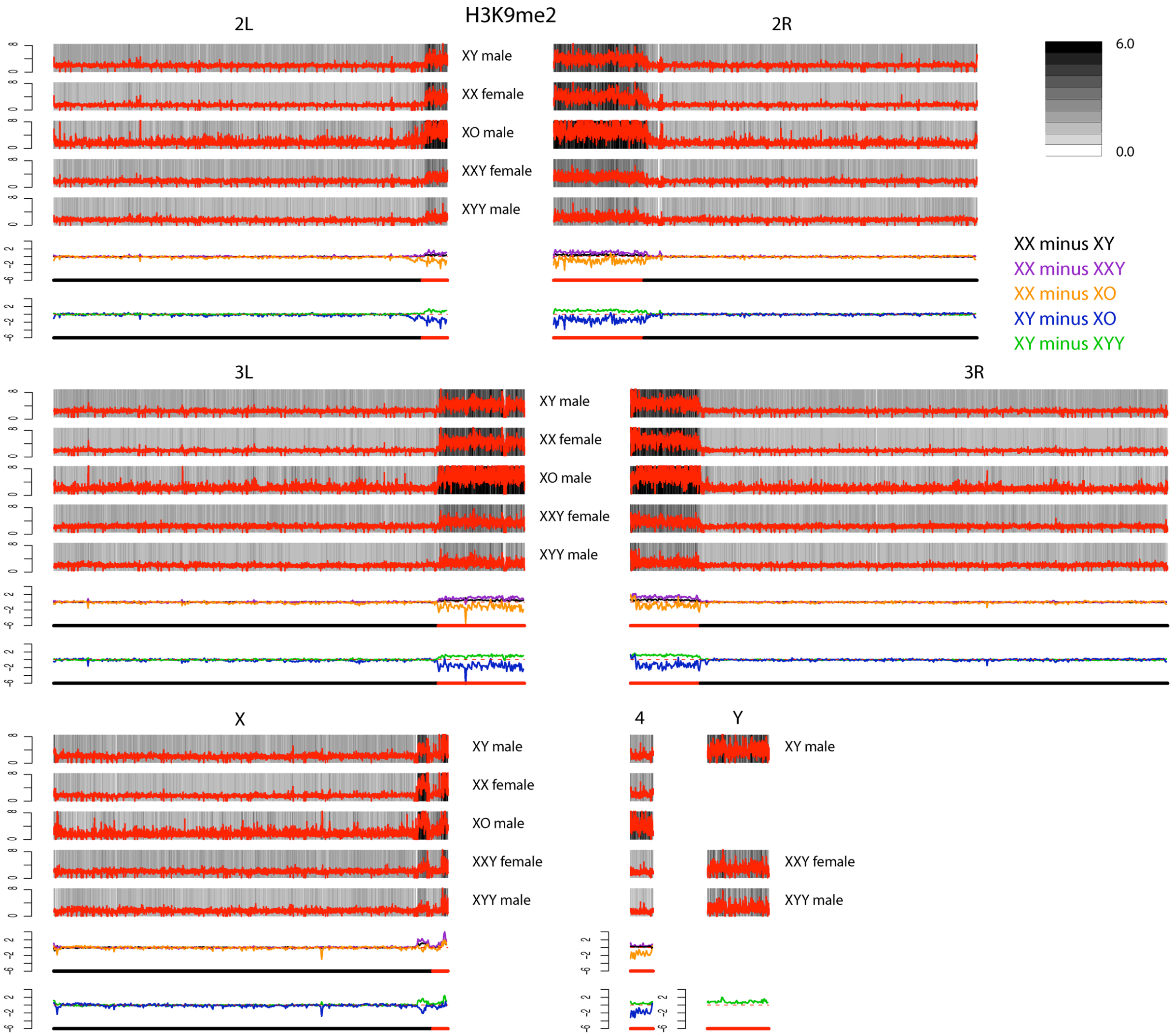

Figure S14. Genome-wide enrichment of H3K9me2 for *D. melanogaster* strains with different karyotypes along the different chromosome arms.

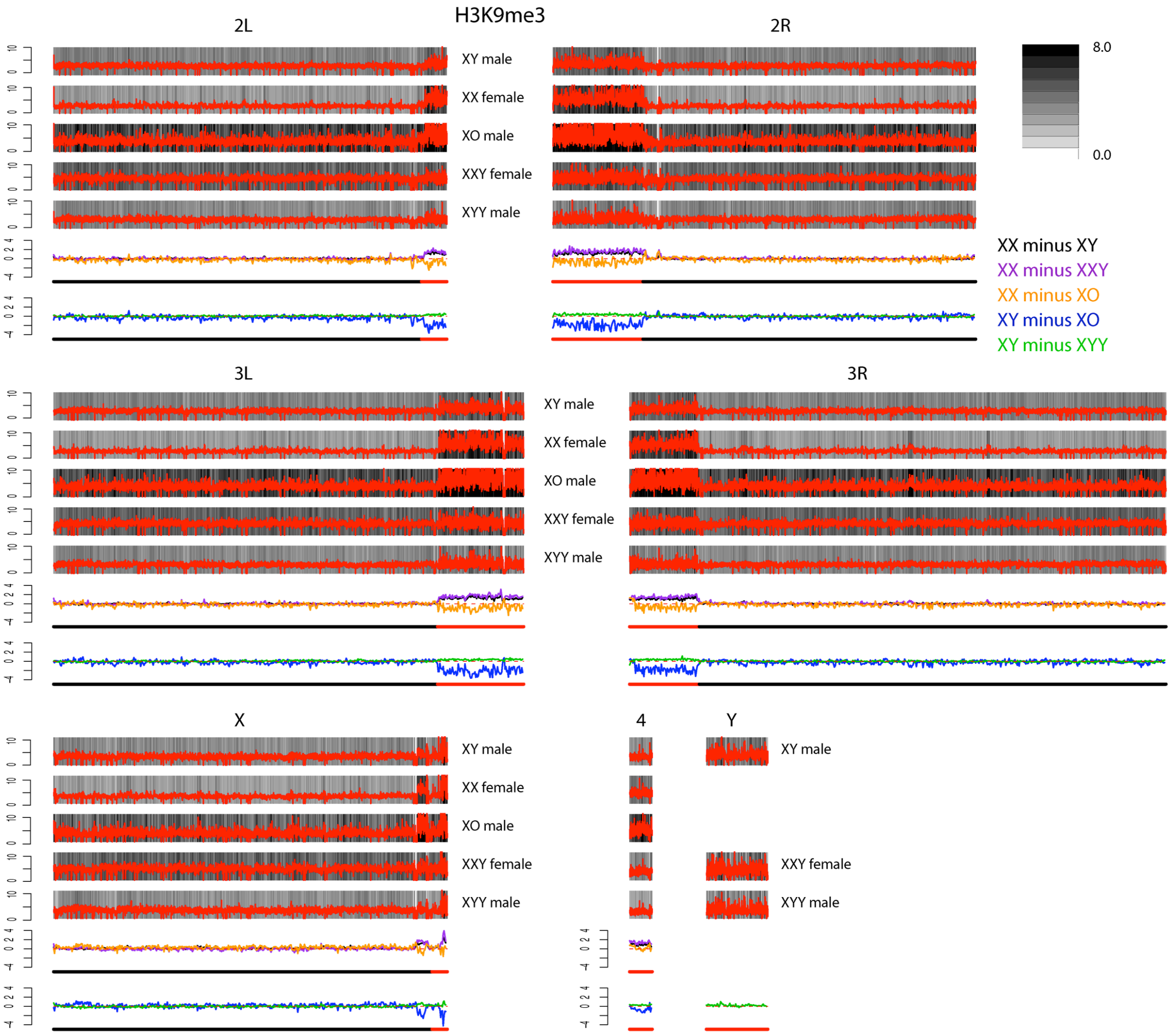

Figure S15. Genome-wide enrichment of H3K9me3 for *D. melanogaster* strains with different karyotypes along the different chromosome arms.

C5. Heterochromatin/euchromatin boundary

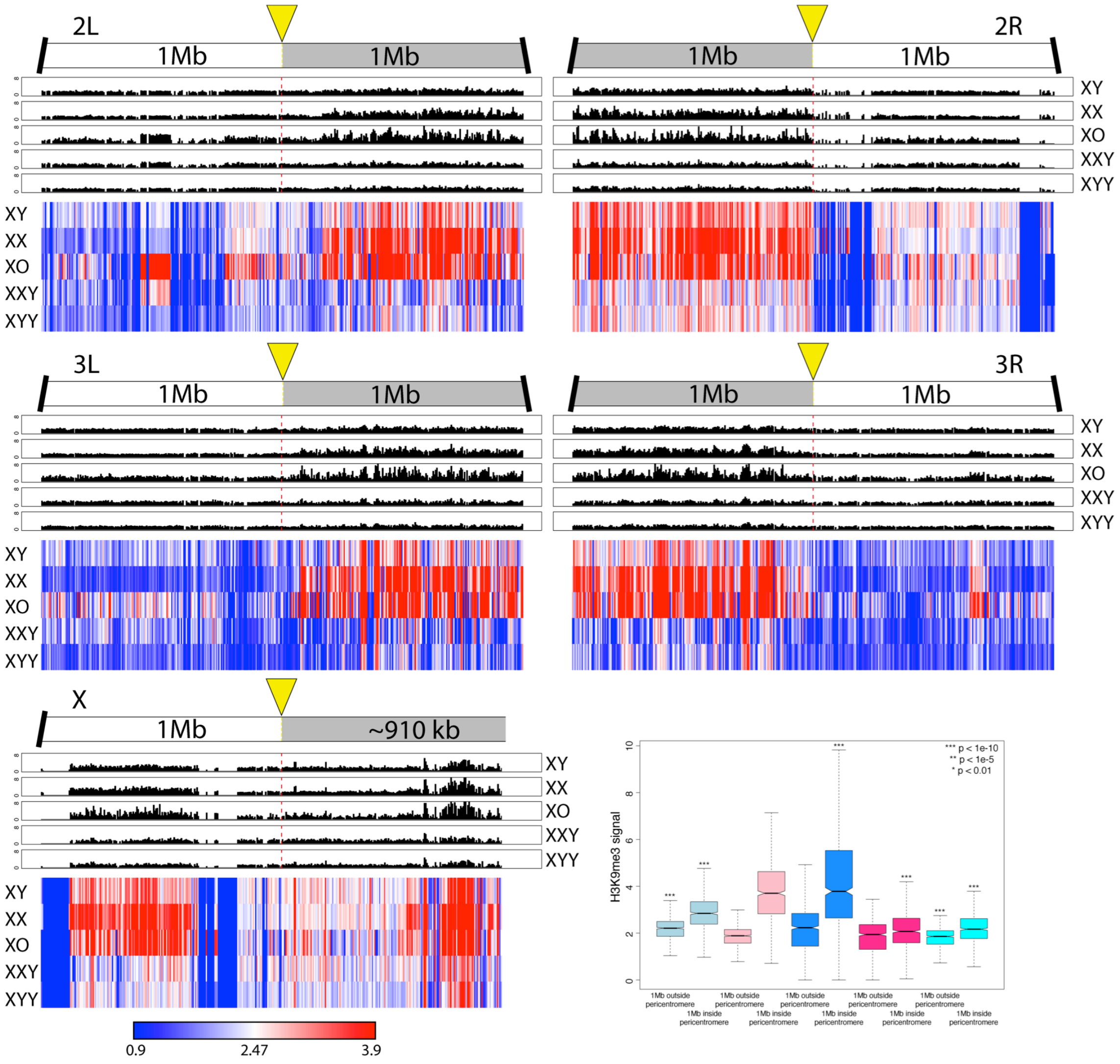

Figure S16. Enrichment of H3K9me3 within 1Mb of the heterochromatin/ euchromatin boundaries. The plots were made in the same manner as for H3K9me2 (see **Figure 5**).

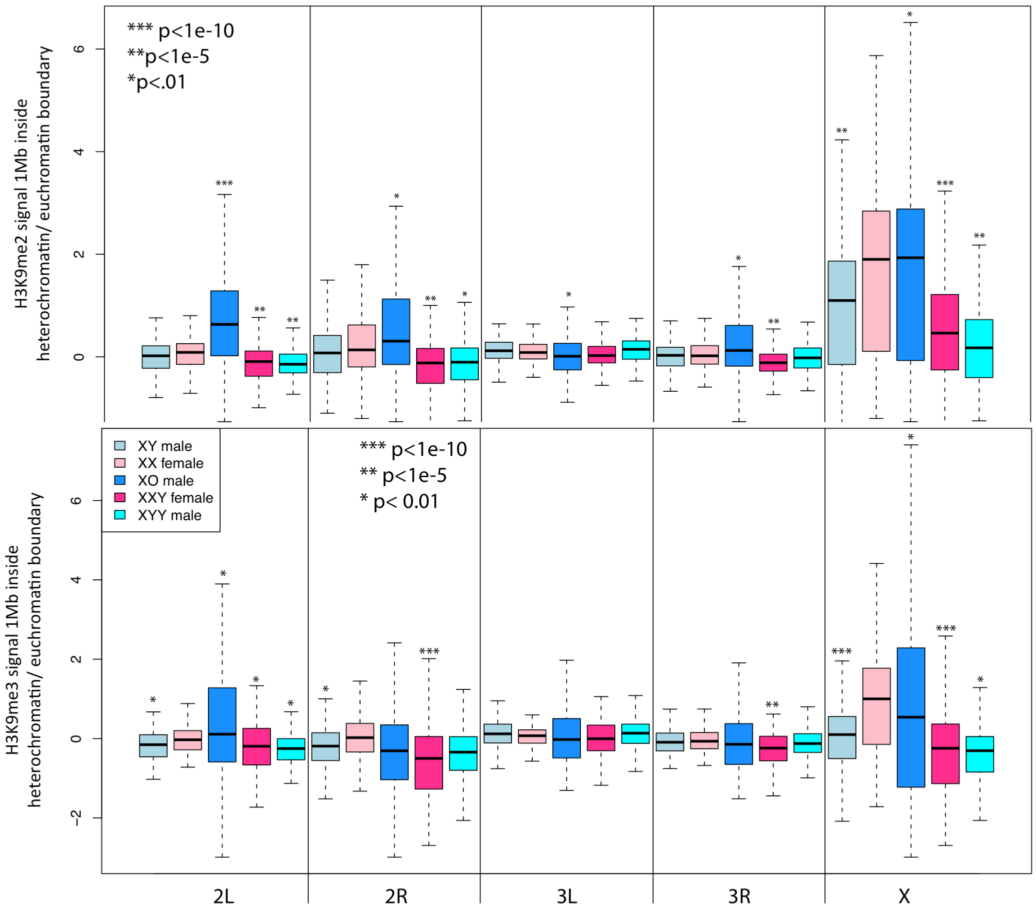

Figure S17. Normalized H3K9me2 and H3K9me3 signal of 5kb windows in euchromatic regions within 1Mb of the heterochromatin/ euchromatin boundary by chromosome arm. Boxes extend from the first to the third quartile and whiskers to the most extreme data point within 1.5 times the interquantile range. Significance values for XY and XXY individuals were calculated relative to XX females, and significance values for XO and XYY individuals were calculated relative to XY males, using the Wilcoxon test.

D. Gene expression

We analyzed replicate RNA-seq data from heads for wildtype males and females, as well as X0, XXY and XYY flies (**Table S7**). Expression values are given in **Appendix 1**.

Table S7. RNA-seq data analyzed

Karyotype	Genotype	Data type	Tissue	Figure	reads generated	SRA accession
XX	Canton-S wt	RNA-seq	head	Fig. 6	13,847,158	TBA
XY	Canton-S wt	RNA-seq	head	Fig. 6	17,655,833	TBA
X0	2549 strain	RNA-seq	head	Fig. 6	16,913,099	TBA
XXY	2549 strain	RNA-seq	head	Fig. 6	6,454,507	TBA
XYY	2549 strain	RNA-seq	head	Fig. 6	16,189,832	TBA
XX	Canton-S wt	RNA-seq	head	Fig. 6	9,072,646	TBA
XY	Canton-S wt	RNA-seq	head	Fig. 6	7,770,905	TBA
X0	2549 strain	RNA-seq	head	Fig. 6	17,961,720	TBA
XXY	2549 strain	RNA-seq	head	Fig. 6	10,034,759	TBA
XYY	2549 strain	RNA-seq	head	Fig. 6	8,315,865	TBA

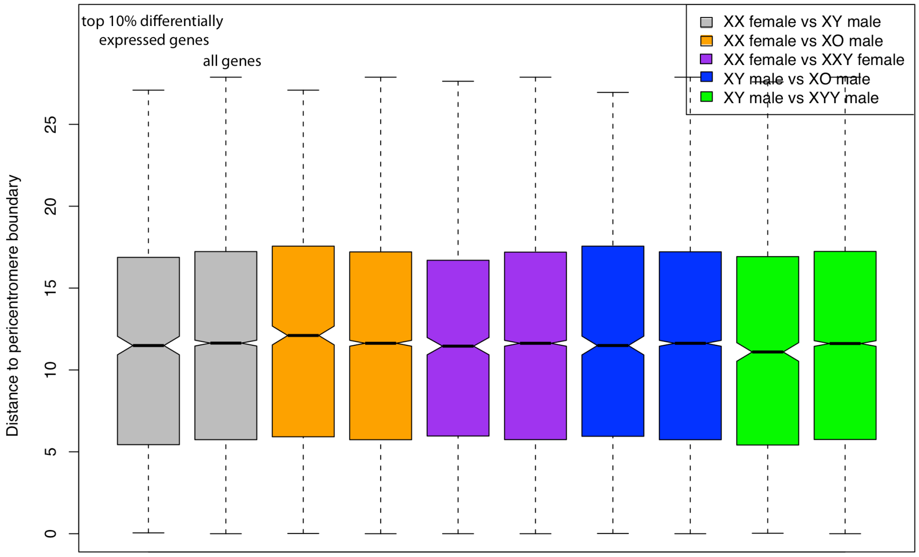

Figure S18. Distance to the pericentromere boundary (in Mb) of the top 10% of differentially expressed genes compared to all genes. For each pair of boxplots, the top 10% of differentially expressed genes are on the left, and all genes are on the right. Boxes extend from the first to the third quartile and whiskers to the most extreme data point within 1.5 times the interquantile range. None of the comparisons are significant (Wilcoxon test).

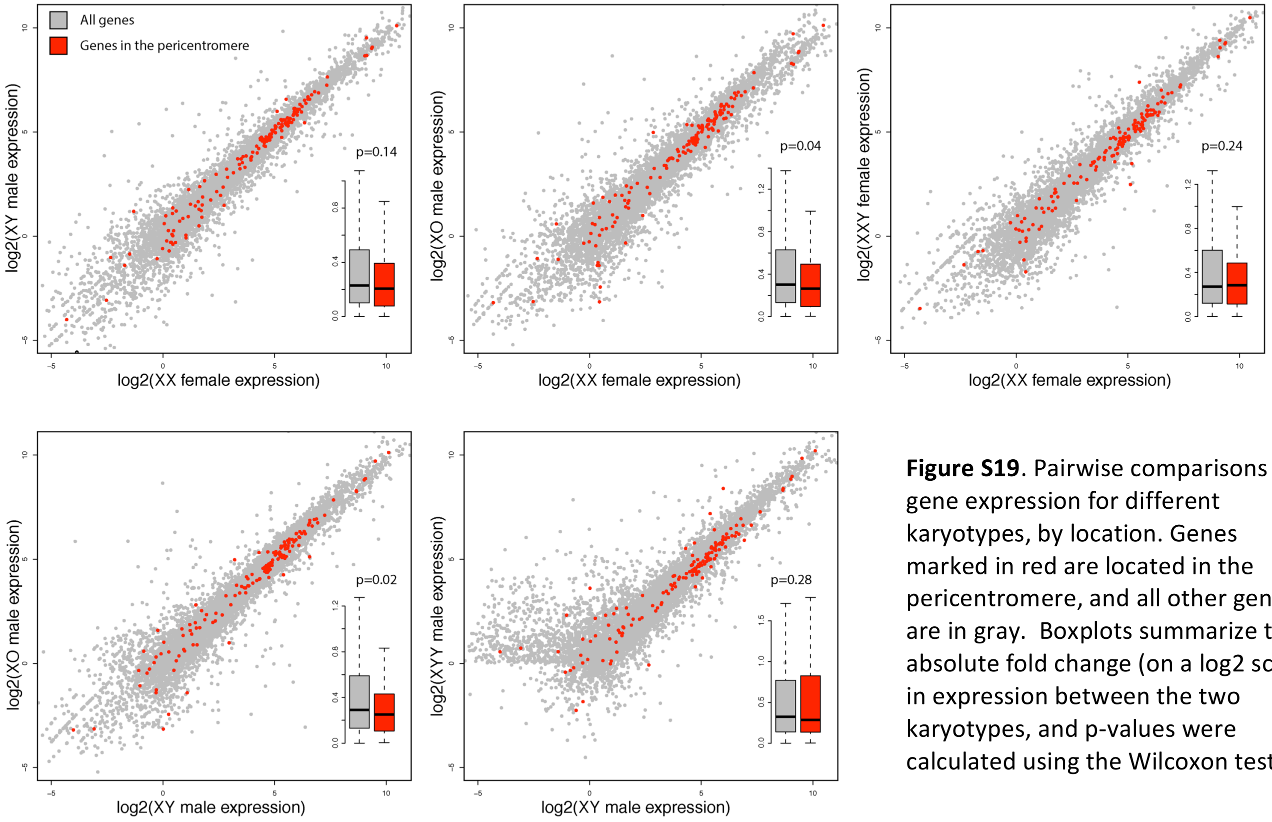

Figure S19. Pairwise comparisons of gene expression for different karyotypes, by location. Genes marked in red are located in the pericentromere, and all other genes are in gray. Boxplots summarize the absolute fold change (on a log2 scale) in expression between the two karyotypes, and p-values were calculated using the Wilcoxon test.

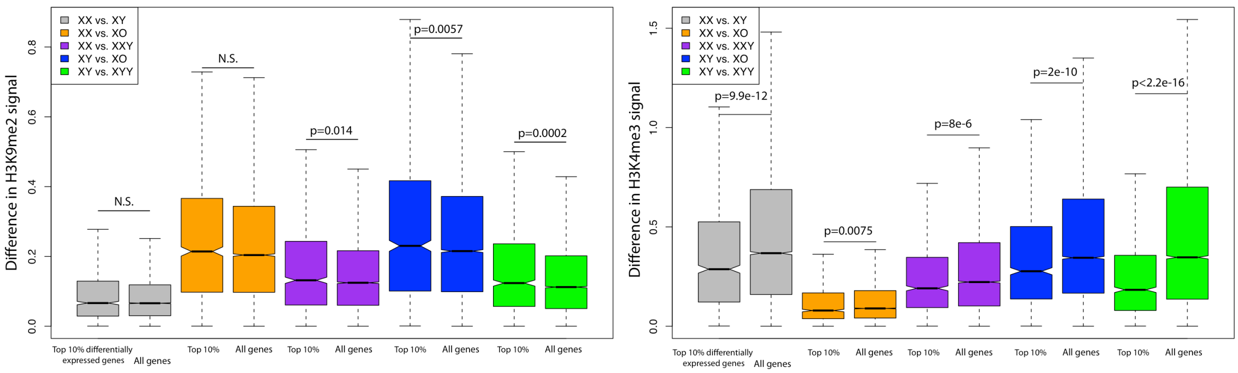

difference in H3K9me2 signal of genes differentially expressed vs. all genes

difference in H3K4me3 signal of genes differentially expressed vs. all genes

Figure S20. Difference in signal of H3K9me2 and H3K4me3 in the top 10% of differentially expressed genes ("Top 10%") compared to all genes for different pairwise comparisons of karyotypes. P-values were calculated for all comparisons using the Wilcoxon test.

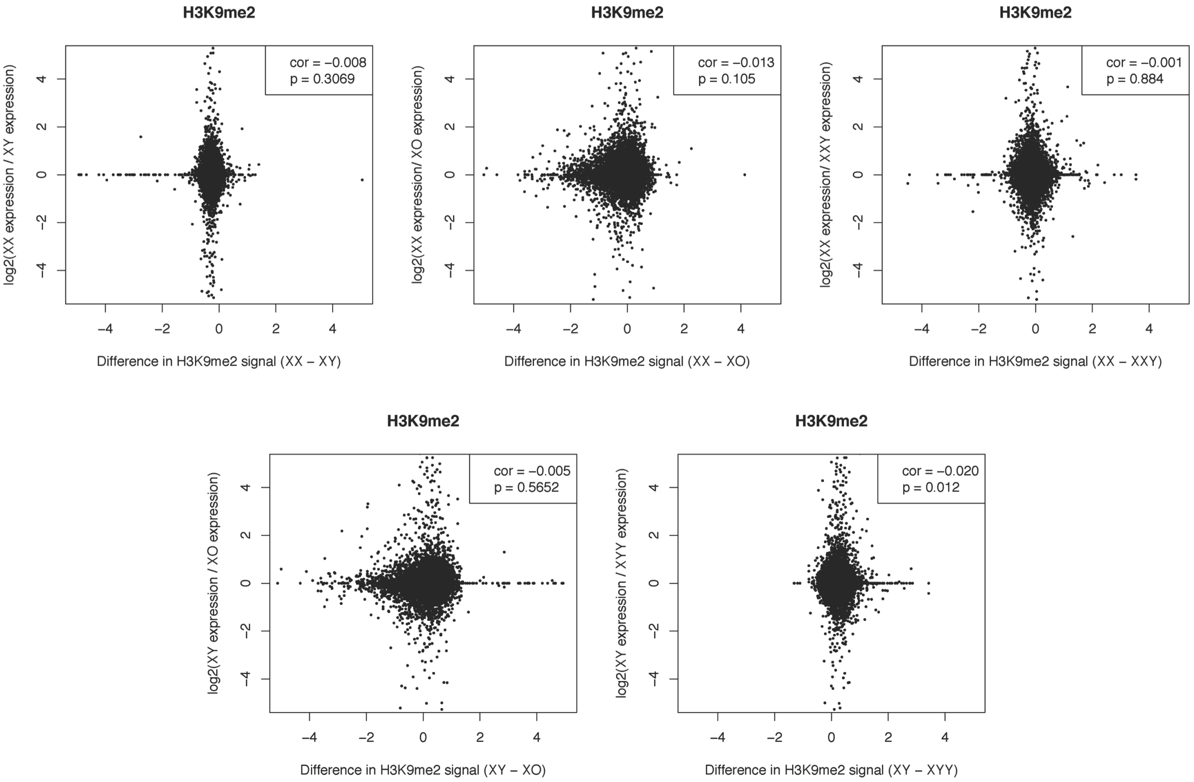

Figure S21. Correlations between expression differences and heterochromatin differences based on H3K9me2 ChIPs for different karyotypes.

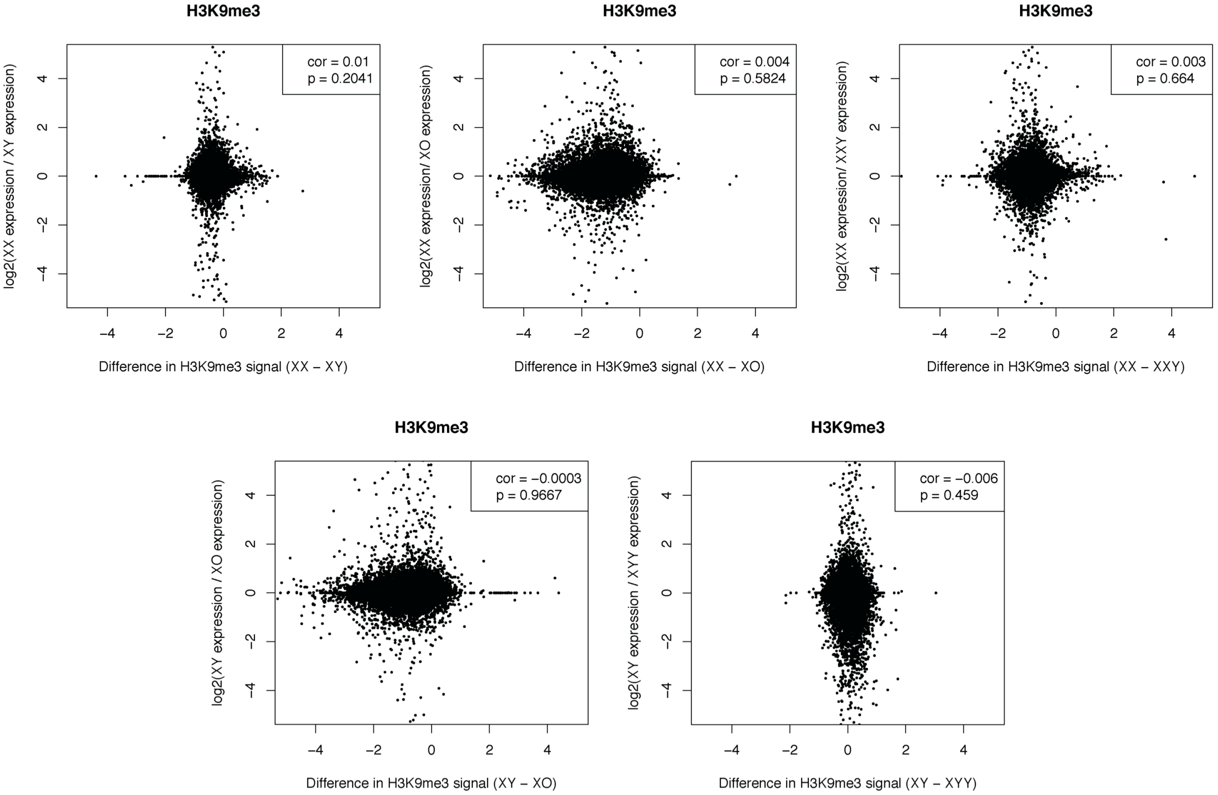

Figure S22. Correlations between expression differences and heterochromatin differences based H3K9me3 ChIPs for different karyotypes.

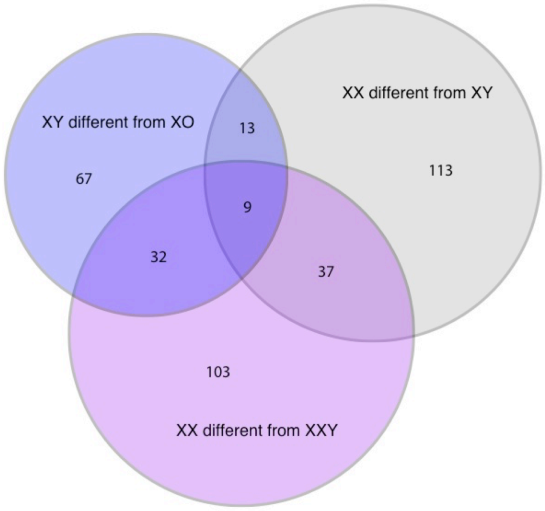

Figure S23. Overlap of genes categorized as significantly differently expressed by Cuffdiff between wildtype XY male and XX female, XX vs. XXY females, and XY vs. XO males.

Table S8. GO categories of differentially expressed genes.

**XX vs. XY**			
**upregulated in XY**		**upregulated in XX**	
Top GO terms	Description	Top GO terms	Description
GO:0051704	multi-organism process	GO:0007548	sex differentiation
GO:0055114	oxidaton-reduction process	GO:0006629	lipid metabolic process
GO:0051707	response to other organism	GO:0016042	lipid catabolic process
GO:0043207	response to external biotic stimulus	GO:0007608	sensory perception of smell
GO:0009607	response to biotic stimulus	GO:0032870	cellular response to hormone stimulus
GO:0034644	cellular response to UV	GO:0007498	mesoderm development
GO:0034462	small-subunit processome assembly	GO:0008286	insulin receptor signaling pathway
GO:0000480	endonucleolytic cleavage in 5-ETS of tricistronic rRNA transcript	GO:0032869	cellular response to insulin stimulus
GO:0000472	endonucleolytic cleavage to generate 5'-end of SSU-rRNA		
GO:0000967	rRNA 5'-end processing		
**XX vs. XO**			
**upregulated in XO**		**upregulated in XX**	
Top GO terms	Description	Top GO terms	Description
GO:0017144	drug metabolic process	GO:0002181	cytoplasmic translation
GO:0040003	chitin-based cuticle development	GO:0043043	peptide biosynthetic process
GO:0042335	cuticle development	GO:0006412	translation
GO:0055114	oxidaton-reduction process	GO:0043604	amide biosynthetic process
GO:0006030	chitin metabolic process	GO:0006518	peptide metabolic process
GO:0042737	drug catabolic process	GO:0043603	cellular amide metabolic process
GO:1901071	glucosamine-containing compound metabolic process	GO:0034645	cellular macromolecule biosynthetic process

GO:0006040	amino sugar metabolic process	GO:1901566	organonitrogen compound biosynthetic process
GO:0044282	small molecule catabolic process	GO:0009059	macromolecule biosynthetic process
GO:0006022	aminoglycan metabolic process	GO:0044271	cellular nitrogen compound biosynthetic process
**XX vs. XXY**			
**upregulated in XXY**		**upregulated in XX**	
Top GO terms	Description	Top GO terms	Description
GO:0006030	chitin metabolic process	GO:0061077	chaperone-mediated protein folding
GO:0017144	drug metabolic process	GO:0042026	protein refolding
GO:1901071	glucosamine-containing compound metabolic process	GO:0051085	chaperone cofactor-dependent protein refolding
GO:0006040	amino sugar metabolic process	GO:0051084	de novo' posttranslational protein folding
GO:0006022	aminoglycan metabolic process	GO:0009408	response to heat
GO:0040003	chitin-based cuticle development	GO:0006458	de novo' protein folding
GO:0042335	cuticle development	GO:0009266	response to temperature stimulus
GO:0042737	drug catabolic process	GO:0006457	protein folding
GO:1901135	carbohydrate derivative metabolic process	GO:0034620	cellular response to unfolded protein
GO:0055114	oxidaton-reduction process	GO:0035080	heat shock-mediated polytein chromosome puffing
**XY vs. XO**			
**upregulated in XO**		**upregulated in XY**	
Top GO terms	Description	Top GO terms	Description
GO:0040003	chitin-based cuticle development	GO:0034605	cellular response to heat
GO:0042335	cuticle development	GO:0009408	response to heat
GO:1901071	glucosamine-containing compound metabolic process	GO:0009266	response to temperature stimulus
GO:0006030	chitin metabolic process	GO:0035080	heat shock-mediated polytene chromosome puffing
GO:0006040	amino sugar metabolic process	GO:0051085	chaperone cofactor-dependent protein refolding
GO:0008365	adult chitin-based cuticle development	GO:0051084	de novo' posttranslational protein folding
GO:0017144	drug metabolic process	GO:0034620	cellular response to unfolded protein
GO:0006022	aminoglycan metabolic process	GO:0006458	de novo' protein folding
GO:0030001	metal ion transport	GO:0035079	polytene chromosome puffing
**XY vs. XYY**			
**upregulated in XYY**		**upregulated in XY**	
Top GO terms	Description	Top GO terms	Description
GO:0032504	multicellular organism reproduction	GO:0009416	response to light stimulus
GO:0000003	reproduction	GO:0007602	phototransduction
GO:0006030	chitin metabolic process	GO:0009583	detection of light stimulus
GO:0017144	drug metabolic process	GO:0071482	cellular response to light stimulus
GO:1901071	glucosamine-containing compound metabolic process	GO:0009582	detection of abiotic stimulus
GO:0006040	amino sugar metabolic process	GO:0009581	detection of external stimulus
GO:0040003	chitin-based cuticle development	GO:0022400	regulation of rhodopsin mediated signaling pathway
GO:0045297	post-mating behavior	GO:0016059	deactivation of rhodopsin mediated signaling
GO:0042737	drug catabolic process	GO:0009628	response to abiotic stimulus
GO:0042335	cuticle development	GO:0009314	response to radiation

E. Repeat Expression

Heterochromatin is established during early embryogenesis to silence repetitive DNA and transposable elements (TEs). **Figures S24-26** show heterochromatin enrichment at TE. Male-specific or male-biased TEs are identified based on coverage from a genome male and female DNA library (**Figure S27**); fasta sequences of these repeat library are given in the **Appendix 2**.

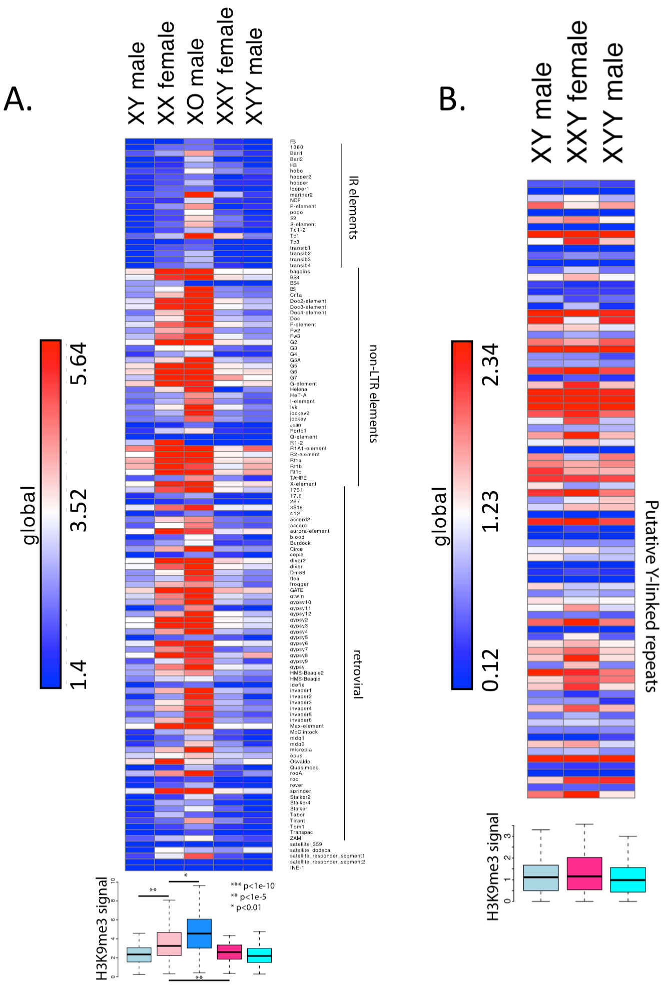

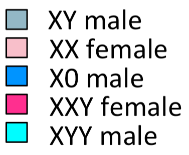

Figure S24. Enrichment of H3K9me3 at different TE families. **A.** All repeats from the library of consensus transposable elements and satellites from FlyBase. Boxplots summarize H3K9me3 signal in these consensus repeats, and p-values were calculated using the Wilcoxon test. **B.** Putatively Y-linked (male-specific) *de novo* assembled repeats only. Boxplots summarize H3K9me3 signal in putatively Y-linked repeats, and p-values were calculated using the Wilcoxon test.

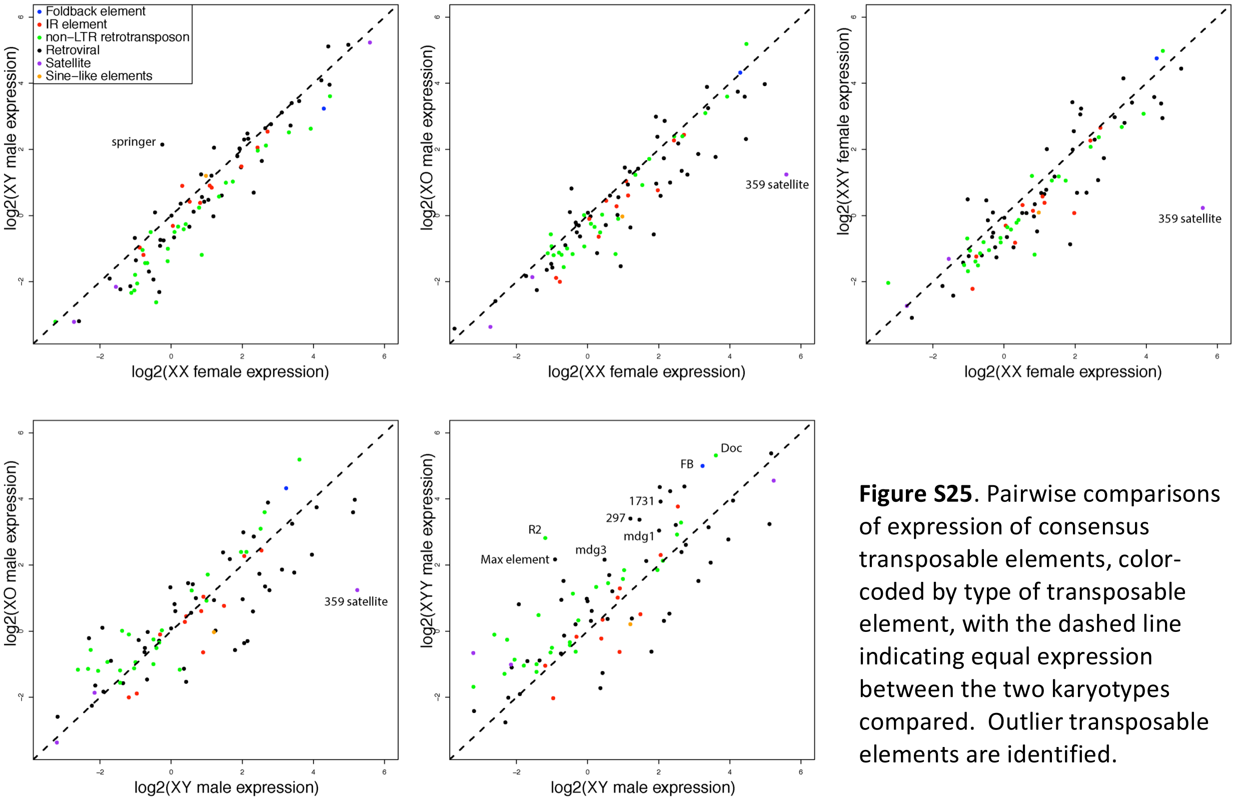

Figure S25. Pairwise comparisons of expression of consensus transposable elements, color-coded by type of transposable element, with the dashed line indicating equal expression between the two karyotypes compared. Outlier transposable elements are identified.

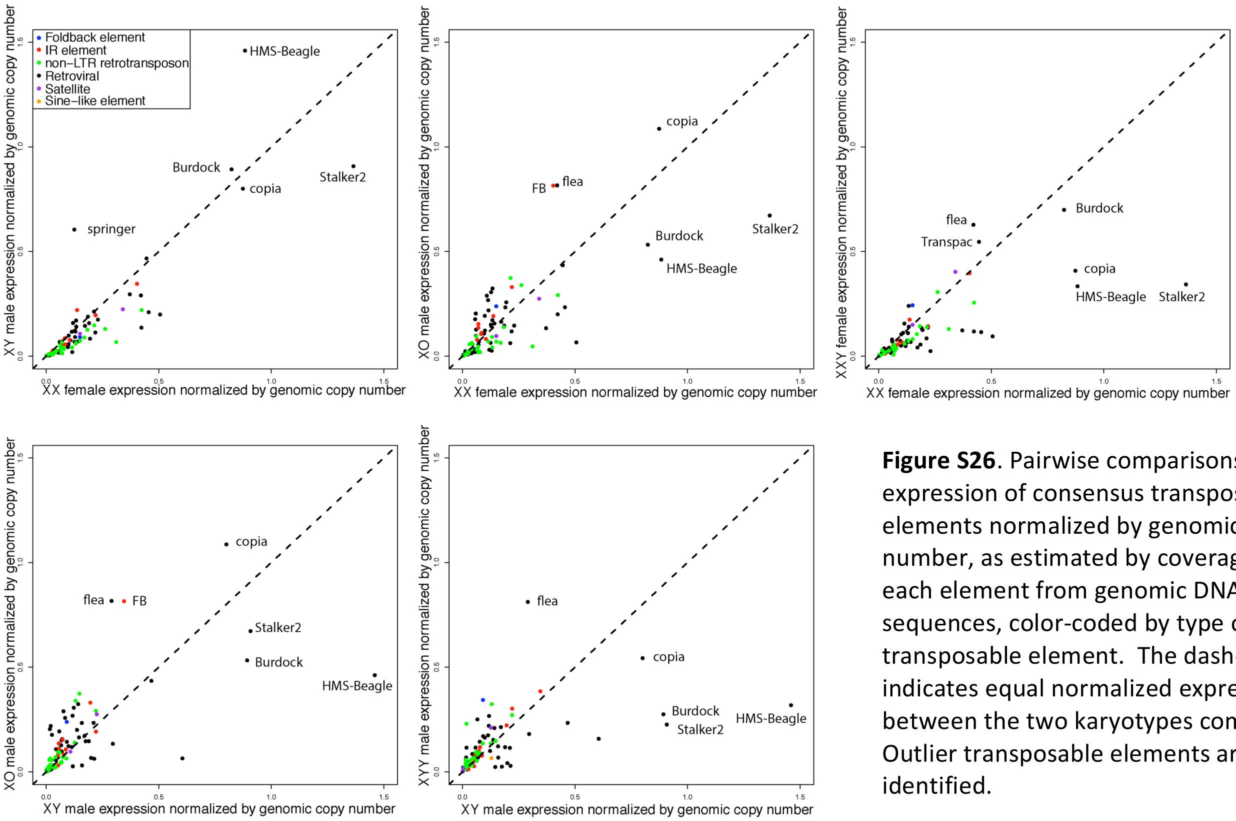

Figure S26. Pairwise comparisons of expression of consensus transposable elements normalized by genomic copy number, as estimated by coverage of each element from genomic DNA sequences, color-coded by type of transposable element. The dashed line indicates equal normalized expression between the two karyotypes compared. Outlier transposable elements are identified.

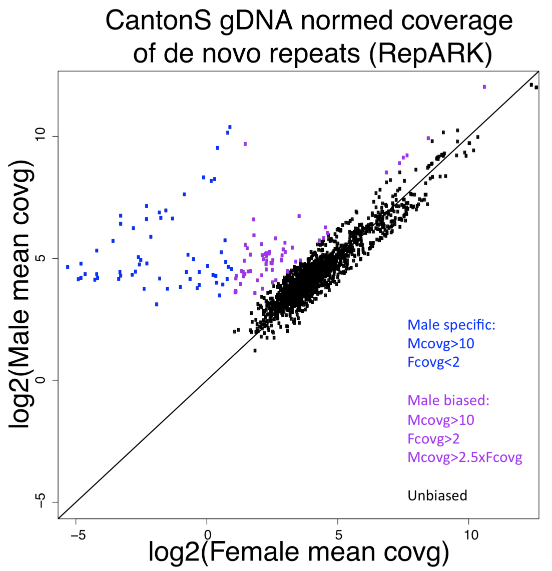

Figure S27. Categorization of *de novo* assembled repeats from RepARK as male-specific, male-biased, or unbiased based on coverage of female and male genomic reads. After *de novo* assembling repeats, we mapped male and female genomic reads to the repeats and removed all repeats that did not have at least 5 times the average genome coverage in one sex. We categorized male-specific repeats as those with at least 10 times the average male genome coverage and less than 2 times the average female genome coverage, and male-biased repeats as those with at least 10 times the average male genome coverage, at least 2 times the average female genome coverage, and male coverage at least 2.5 times higher than female coverage (Figure taken from Brown et al submitted).

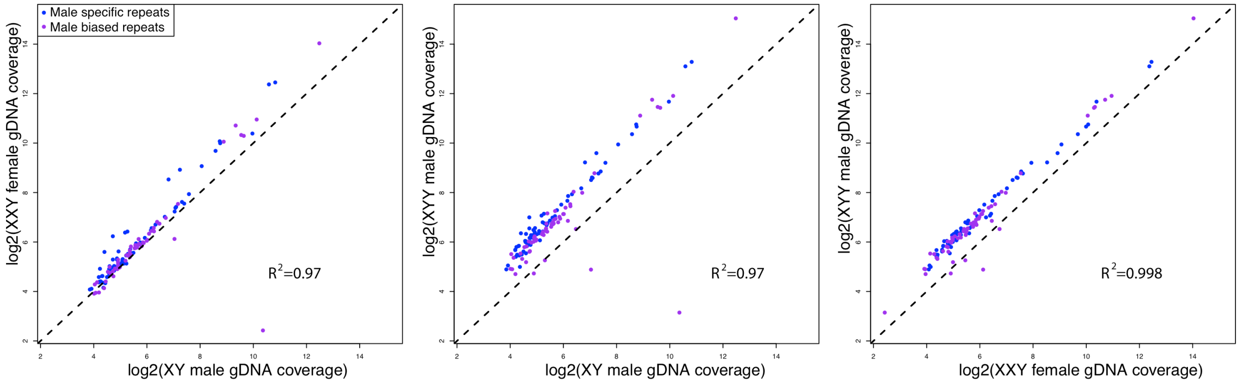

Figure S28. Pairwise comparisons of genomic coverage of *de novo* assembled male-biased and male-specific repeats for karyotypes containing at least one Y chromosome (XY, XXY, and XYY). Genomic coverage is estimated from sequencing reads of libraries constructed from genomic DNA of XY, XXY and XYY flies. The dashed line indicates equal coverage of repeats in the karyotypes compared.

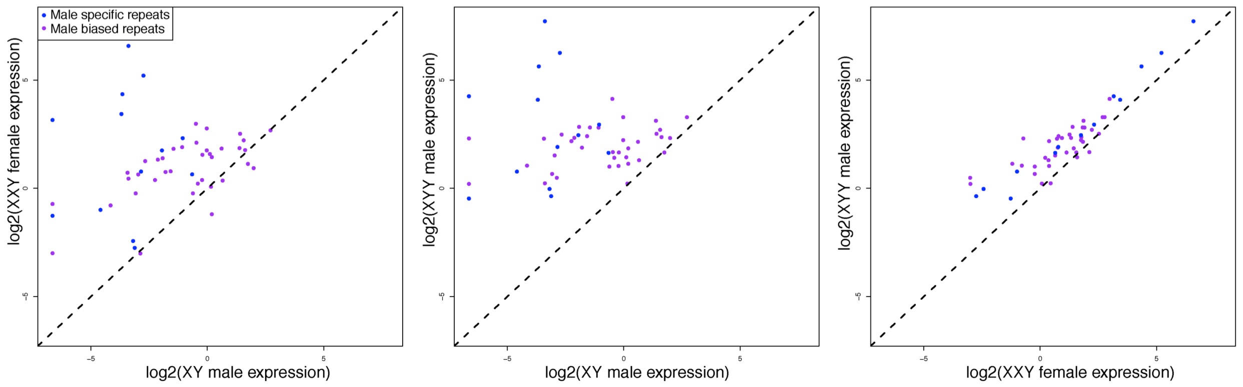

Figure S29. Pairwise comparisons of expression of *de novo* assembled male-biased and male-specific repeats for karyotypes containing at least one Y chromosome (XY, XXY, and XYY). Expression levels are not corrected by genomic copy number, instead reflecting the total number of transcripts from putatively Y-linked repeats in each karyotype.

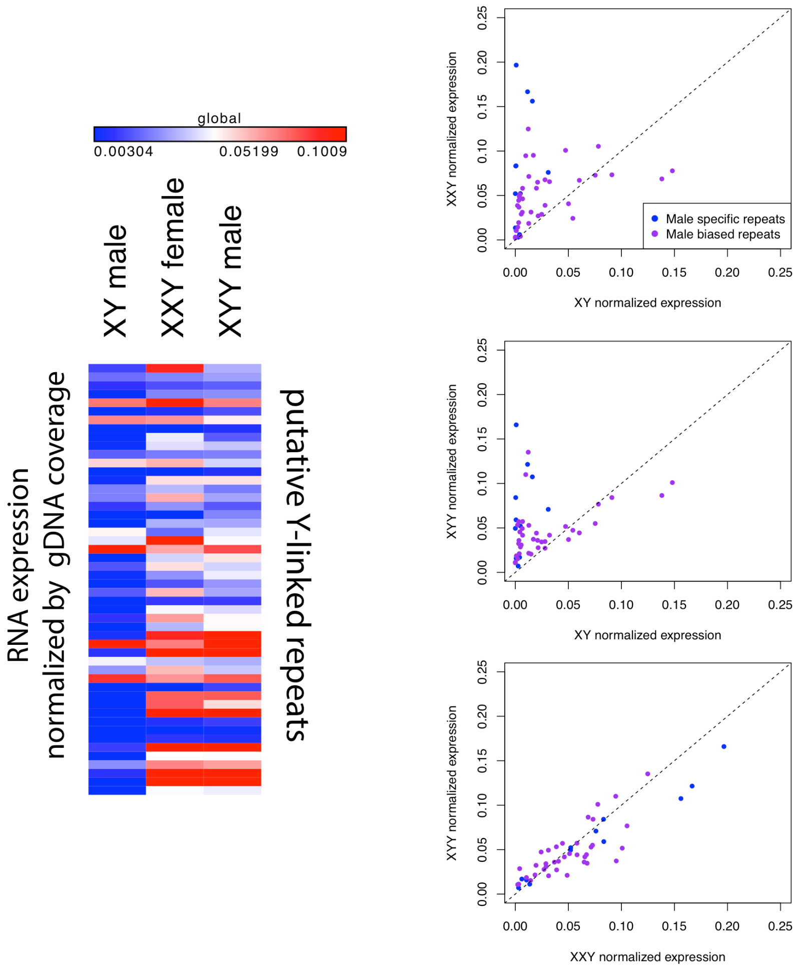

Figure S30. Expression of male-specific/ male-biased repeats normalized by genomic copy number in karyotypes with at least one Y chromosome (XY, XXY, and XYY). The heatmap indicates the 90th percentile or higher values in deep red, the 10th percentile or lower values in deep blue, across all karyotypes and repeats. The scatterplots show pairwise comparisons of expression values normalized by genomic coverage, with the dashed line indicating equal expression between the two karyotypes compared.
